# Iron deficiency in non-pregnant women with normal hemoglobin: a cross-sectional analysis of risk factors and clinical implications

**DOI:** 10.3389/fmed.2025.1700235

**Published:** 2026-01-23

**Authors:** Nawal Alsubaie, Zafar Ali Shah, Muhammad Ilyas, Shafqat Ayaz, Ijaz Habib, Ammena Y. Binsaleh, Amani S. Alrossies

**Affiliations:** 1 Department of Pharmacy Practice, College of Pharmacy, Princess Nourah Bint Abdulrahman University, Riyadh, Saudi Arabia; 2 Department of Agricultural Chemistry and Biochemistry, Faculty of Nutrition Sciences, The University of Agriculture, Peshawar, Khyber Pakhtunkhwa, Pakistan; 3 Department of Nutrition, Northwest General Hospital and Research Centre, Peshawar, Khyber Pakhtunkhwa, Pakistan; 4 Institute of Public Health and Social Sciences, Khyber Medical University, Peshawar, Khyber Pakhtunkhwa, Pakistan; 5 United Nations World Food Programme, University Town, Peshawar, Khyber Pakhtunkhwa, Pakistan

**Keywords:** associations, breastfeeding, hemoglobin, iron deficiency without anemia, non-pregnant women, reproductive health, serum ferritin

## Abstract

**Background:**

Iron deficiency without anemia (IDWA) is a widespread but underdiagnosed condition in women of reproductive age. Traditional screening approaches that rely solely on hemoglobin levels may overlook significant iron depletion in seemingly healthy female patients, leading to missed opportunities for intervention against impaired cognition, reduced exercise capacity, and an increased risk of infection in this population.

**Objective:**

The objective of the study was to determine the prevalence of iron deficiency in non-pregnant women with normal hemoglobin levels and to identify the factors associated with iron deficiency.

**Methods:**

This cross-sectional study was conducted at an urban teaching hospital between March and August 2024. A total of 127 non-pregnant women aged 16–45 years were eligible; 100 women with hemoglobin levels ≥11 g/dL were enrolled using consecutive sampling (refusal rate: 21.3%, *n* = 27). Data on demographic characteristics, clinical history, reproductive factors, dietary habits, and laboratory evaluations (hemoglobin, serum ferritin, and serum iron levels) were collected for each patient. Iron deficiency was defined as a serum ferritin level of <15 μg/L or a serum iron level of <10 μmol/L. A multivariable logistic regression analysis was adjusted for age, BMI, parity, dietary patterns, physical activity, and iron supplementation.

**Results:**

Iron deficiency was identified in 41% (41/100) of the participants despite normal hemoglobin levels. A univariate analysis revealed significant associations between iron deficiency and a history of breastfeeding (crude OR = 9.13, 95% CI: 3.32–25.11, *p* < 0.001) and of anemia (crude OR = 5.51, 95% CI: 2.10–14.46, p < 0.001). After multivariable adjustment, breastfeeding history remained strongly associated with iron deficiency (adjusted OR = 6.72, 95% CI: 2.01–22.44, *p* = 0.002), as well as with history of anemia (adjusted OR = 4.92, 95% CI: 1.56–15.53, *p* = 0.007). Underweight BMI showed an elevated point estimate but did not reach statistical significance (adjusted OR = 2.84, 95% CI: 0.68–11.83, *p* = 0.154), likely due to the limited sample size in this subgroup. Model diagnostics were satisfactory (Hosmer–Lemeshow *χ*^2^ = 3.21, *p* = 0.921; all variance inflation factors (VIFs) < 2.0).

**Conclusion:**

A substantial proportion of non-pregnant women with normal hemoglobin levels demonstrated biochemical evidence of iron deficiency. Breastfeeding history and a history of anemia were the strongest independent factors associated with iron deficiency. A comprehensive iron assessment, including ferritin and serum iron levels, is recommended for at-risk populations, particularly those with a history of breastfeeding or anemia.

## 1 Introduction

Iron deficiency remains one of the most common micronutrient deficiencies worldwide, affecting an estimated 1.2 billion individuals globally, with a particularly high prevalence among women of reproductive age (1). In women, iron deficiency is driven by multiple factors, including menstrual blood loss, dietary insufficiency, malabsorption, and reproductive demands, such as pregnancy and lactation (2).

Traditional clinical approaches predominantly rely on hemoglobin concentration to assess iron status and diagnose anemia. However, this practice overlooks a substantial proportion of individuals with depleted iron stores but preserved hemoglobin levels, a condition termed iron deficiency without anemia (IDWA) (3). The WHO recognizes IDWA as a distinct clinical entity with significant health implications, including impaired cognition, reduced exercise capacity, and an increased risk of infection (4).

The diagnostic gap regarding IDWA is particularly pronounced in women in low- and middle-income countries, where iron assessment beyond hemoglobin is often limited by resource constraints and the lack of systematic screening protocols. This limitation is clinically important because early detection of iron depletion in non-anemic women provides an opportunity for preventive intervention before progression to anemia (5). In Pakistan, where iron deficiency anemia remains a significant public health challenge, understanding the preanemic stage is crucial for targeted interventions (6).

Previous epidemiological studies have characterized iron deficiency in pregnant women and women with established anemia; however, comparative data on iron status, specifically in non-pregnant women with normal hemoglobin levels, are sparse, particularly in the context of Pakistan and South Asia. Understanding the prevalence and determinants of IDWA in this population is essential for refining clinical screening guidelines and public health interventions (7, 8).

This study aimed to determine the prevalence of iron deficiency in non-pregnant women with normal hemoglobin levels and to identify demographic, clinical, reproductive, and lifestyle factors associated with iron deficiency in this group.

## 2 Methods

### 2.1 Study design and setting

This cross-sectional analytical study was conducted in the outpatient department of an urban teaching hospital in Peshawar, Pakistan, between March and August 2024. The hospital serves a mixed urban and peri-urban population. The study was approved by the Institutional Review Board (Ref: IRB/2024/034) and adhered to the Declaration of Helsinki.

### 2.2 Study sampling and participants

During the 6-month study period, 127 non-pregnant women aged 16–45 years attending the hospital outpatient department met the initial eligibility criteria. Using consecutive sampling, 100 women were enrolled; 27 declined to participate (refusal rate: 21.3%) due to time constraints (*n* = 15), reluctance to provide blood samples (*n* = 8), or undisclosed reasons (*n* = 4). A sample size of 100 was determined to detect a 15% difference in the prevalence of iron deficiency (assuming a baseline prevalence of 20% and detecting 35% in an exposed group) with 80% power and a two-sided alpha of 0.05. Additionally, this sample size provided 80% power to detect an odds ratio of approximately 2.5 for a predictor with 20% prevalence, assuming an iron deficiency prevalence of 40% in the unexposed group.

A flow diagram detailing the participant recruitment, screening, and enrollment processes is provided in Supplementary Figure S1.

The selection of participants was guided by specific inclusion and exclusion criteria to ensure a homogeneous study population.

### 2.3 Inclusion criteria

Women who were not pregnant, aged between 16 and 45 years, with a hemoglobin level of at least 11 g/dL, and who were willing to provide written informed consent were included in the study.

### 2.4 Exclusion criteria

The exclusion criteria were as follows: pregnant or lactating women, to mitigate the confounding effects of physiological changes; hemoglobin levels below 11 g/dL, to ensure that the participants were not already anemic; a self-reported history of chronic inflammatory disorders; recent iron supplementation within the past 3 months; chronic kidney or liver disease; and active malignancy.

### 2.5 Data collection

Demographic data collected included age, urban or rural residence, education level, marital status, and occupation of the participants. Prior anemia was defined as a self-reported physician diagnosis or a documented history of hemoglobin levels of < 11 g/dL, as recalled by the participant.

### 2.6 Reproductive and menstrual history

For non-pregnant, non-postpartum women (*n* = 93), menstrual regularity, presence of heavy menstrual bleeding (subjective self-report), and contraceptive use were also recorded. Among all participants, the following were documented: parity (number of prior deliveries), postpartum status (within 6 months after delivery), and breastfeeding history (ever breastfed and currently breastfeeding).

### 2.7 Lifestyle and dietary assessment

Physical activity was categorized as “active” or “sedentary” based on self-reported activities. Meat consumption frequency was recorded as “<3 times per week” or “≥3 times per week” as a proxy for the dietary intake of heme iron. A more comprehensive dietary assessment using validated food frequency questionnaires was not feasible because of time and resource constraints within the study design. Current iron supplementation was also noted.

### 2.8 Informed consent

All participants provided written informed consent. Informed consent forms were available in Urdu (the local language) and English. Research staff verbally reviewed key study information with each participant to ensure understanding, regardless of literacy level. None of the participants reported any difficulty in understanding the consent process.

### 2.9 Laboratory analysis

Venous blood samples (5 mL) were collected in the morning after an 8-h overnight fast. The samples were processed within 2 h at the hospital’s centralized laboratory using standardized protocols.

#### 2.9.1 Hemoglobin levels (g/dL)

The data were quantified using an automated hematology analyzer (Cell-Dyn 3,700; Abbott Diagnostics).

#### 2.9.2 Serum ferritin (μg/L)

The results were quantified using a chemiluminescence immunoassay (Abbott Architect).

#### 2.9.3 Serum Iron (μmol/L)

It was measured using a colorimetric assay on an automated chemistry analyzer.

All measurements were performed by laboratory technicians who were blinded to the participants’ demographic and clinical characteristics, thereby reducing measurement bias.

#### 2.9.4 Outcome definition

Iron deficiency was defined as serum ferritin <15 μg/L or serum iron <10 μmol/L, consistent with the WHO criteria. This dual-marker approach was used to enhance diagnostic sensitivity, as these biomarkers provide complementary information on iron stores and circulating iron.

### 2.10 Statistical analysis

#### 2.10.1 Descriptive analysis

Continuous variables were summarized using the mean (SD) or median (IQR), depending on the distributional properties, and categorical variables were presented as frequencies and percentages.

#### 2.10.2 Univariate (crude) analysis

Binary logistic regression analysis was used to calculate crude odds ratios (OR) with 95% confidence intervals (CI) for each iron deficiency predictor, with *p*-values derived from Wald’s test.

#### 2.10.3 Multivariate (adjusted) analysis

A multivariable logistic regression model was constructed with iron deficiency as a binary outcome variable. Variables were selected for inclusion based on *a priori* clinical importance and prior literature: age (continuous), BMI category (normal as reference), anemia history (yes/no), breastfeeding history (yes/no), hypertension (yes/no), lifestyle (sedentary vs. active), iron supplementation use (yes/no), parity (number of deliveries and continuous), and meat intake (<3 times/week vs. ≥3 times/week).

#### 2.10.4 Variables not included in the final model

Variables were selected for inclusion based on *a priori* clinical importance and prior literature. Variables not included in the final model either demonstrated weak bivariate associations (*p* > 0.50, e.g., contraceptive use, retained in the crude analysis only for transparency), were deemed unreliable for precise measurement (e.g., subjective menstrual blood loss not via a validated tool), or showed insufficient variance within the study population (e.g., socioeconomic status, as assessed by education level, which showed limited variability across participants in this urban setting).

#### 2.10.5 Model diagnostics

Goodness-of-fit was assessed using the Hosmer–Lemeshow test (target *p* > 0.05). Multicollinearity was evaluated using variance inflation factors (VIF; all values retained <2.0). Model discrimination was assessed using receiver operating characteristic (ROC) analysis.

#### 2.10.6 Sensitivity analyses

Subgroup analyses were conducted among women with a history of breastfeeding (*n* = 58) and those with a history of anemia (*n* = 27). The analysis was repeated, excluding postpartum women (*n* = 7), to evaluate the stability of the findings.

#### 2.10.7 Post-hoc power analysis

For the underweight BMI subgroup (*n* = 15), post-hoc power was calculated for the observed effect size (adjusted OR = 2.84) at *α* = 0.05, yielding a power of 0.62.

All analyses were performed using R version 4.3.1^1^ with the tidyverse, broom, epitools, and car packages. Statistical significance was defined as a two-tailed *p*-value of <0.05.

## 3 Results

### 3.1 Study population and sampling characteristics

Among the 127 eligible women approached, 100 participated (response rate 78.7%). The demographic characteristics are presented in Table 1. The mean age was 31.2 (SD = 9.4) years. The majority of women (62%) were urban residents, 69% were married, and 42% had completed secondary education. Seven women (7%) were in the postpartum period (≤6 months post-delivery), and 28 (28%) reported that they were currently breastfeeding.

**Table 1:** Demographic and clinical characteristics of study participants stratified by iron deficiency status (*N* = 100).

Variable	Category	Total	Negative	Positive	*p*-value
Age		30.98 (7.97)	30.10 (8.10)	32.24 (7.72)	0.184
BMI		23.88 (4.99)	24.42 (4.82)	23.11 (5.19)	0.207
Number of deliveries		1.40 (1.40)	1.19 (1.33)	1.71 (1.45)	0.072
BMI category	Normal	54 (54.0%)	33 (55.9%)	21 (51.2%)	0.256
	Overweight/obese	31 (31.0%)	20 (33.9%)	11 (26.8%)	
	Underweight	15 (15.0%)	6 (10.2%)	9 (22.0%)	
Residence	Rural	43 (43.0%)	28 (47.5%)	15 (36.6%)	0.280
	Urban	57 (57.0%)	31 (52.5%)	26 (63.4%)	
Marital status	Married	81 (81.0%)	43 (72.9%)	38 (92.7%)	0.013
	Unmarried	19 (19.0%)	16 (27.1%)	3 (7.3%)	
History of anemia	No	73 (73.0%)	51 (86.4%)	22 (53.7%)	<0.001
	Yes	27 (27.0%)	8 (13.6%)	19 (46.3%)	
Breastfeeding history	No	42 (42.0%)	36 (61.0%)	6 (14.6%)	<0.001
	Yes	58 (58.0%)	23 (39.0%)	35 (85.4%)	
Hypertension	No	79 (79.0%)	49 (83.1%)	30 (73.2%)	0.233
	Yes	21 (21.0%)	10 (16.9%)	11 (26.8%)	
Diabetes	No	87 (87.0%)	49 (83.1%)	38 (92.7%)	0.159
	Yes	13 (13.0%)	10 (16.9%)	3 (7.3%)	
Lifestyle	Active	45 (45.0%)	25 (42.4%)	20 (48.8%)	0.526
	Sedentary	55 (55.0%)	34 (57.6%)	21 (51.2%)	
Iron supplement use	No	60 (60.0%)	34 (57.6%)	26 (63.4%)	0.561
	Yes	40 (40.0%)	25 (42.4%)	15 (36.6%)	
Meat intake	<3 times/week	64 (64.0%)	35 (59.3%)	29 (70.7%)	0.242
	≥3 times/week	36 (36.0%)	24 (40.7%)	12 (29.3%)	

Data are presented as mean (SD) for continuous variables and *n* (%) for categorical variables. *P*-values were calculated using *t*-tests for continuous variables and the chi-square or Fisher’s exact tests for categorical variables.

Grouped by iron deficiency status, mean ages were similar between the groups (negative: 31.5 ± 9.8 years vs. positive: 30.7 ± 9.1 years, *p* = 0.760). The BMI showed no statistically significant difference (negative: 23.8 ± 3.2 vs. positive: 24.1 ± 3.5, *p* = 0.158), although the prevalence of underweight participants was slightly higher in the iron deficiency-positive group (53.3% vs. 44.4%).

Clinical history showed that 27% of patients reported prior anemia, 21% had hypertension, and 13% had diabetes. Among the reproductive factors, 58% had ever breastfed (30/58 = 51.7% with iron deficiency vs. 11/42 = 26.2% without). Lifestyle assessments revealed that 55% of the participants reported sedentary activity. A total of 40% of participants used iron supplements at the time of enrollment. Overall, 64% of the participants consumed meat <3 times per week.

Following the description of our study population, we present the demographic and clinical characteristics of the participants, further stratified by their iron deficiency status (Table 2).

**Table 2:** Laboratory parameters (hemoglobin, serum ferritin, and serum iron) stratified by iron deficiency status (*N* = 100).

Parameters	Total	Negative	Positive	*p*-value
Hemoglobin (g/dL)	12.84 (0.92)	12.83 (0.80)	12.85 (1.09)	0.942
Serum ferritin (μg/L)	36.40 (14.07–64.75)	56.70 (40.95–85.05)	12.70 (8.80–16.00)	0.000
Serum iron (μmol/L)	14.05 (9.60–22.27)	21.40 (14.80–25.25)	8.10 (6.30–11.60)	0.000

Hemoglobin is presented as mean (SD); ferritin and serum iron are presented as median (IQR) due to skewed distributions. *P*-values for hemoglobin were calculated using the *t*-test; *p*-values for ferritin and serum iron were calculated using the Wilcoxon rank-sum test.

### 3.2 Iron deficiency prevalence

**Figure 1:**
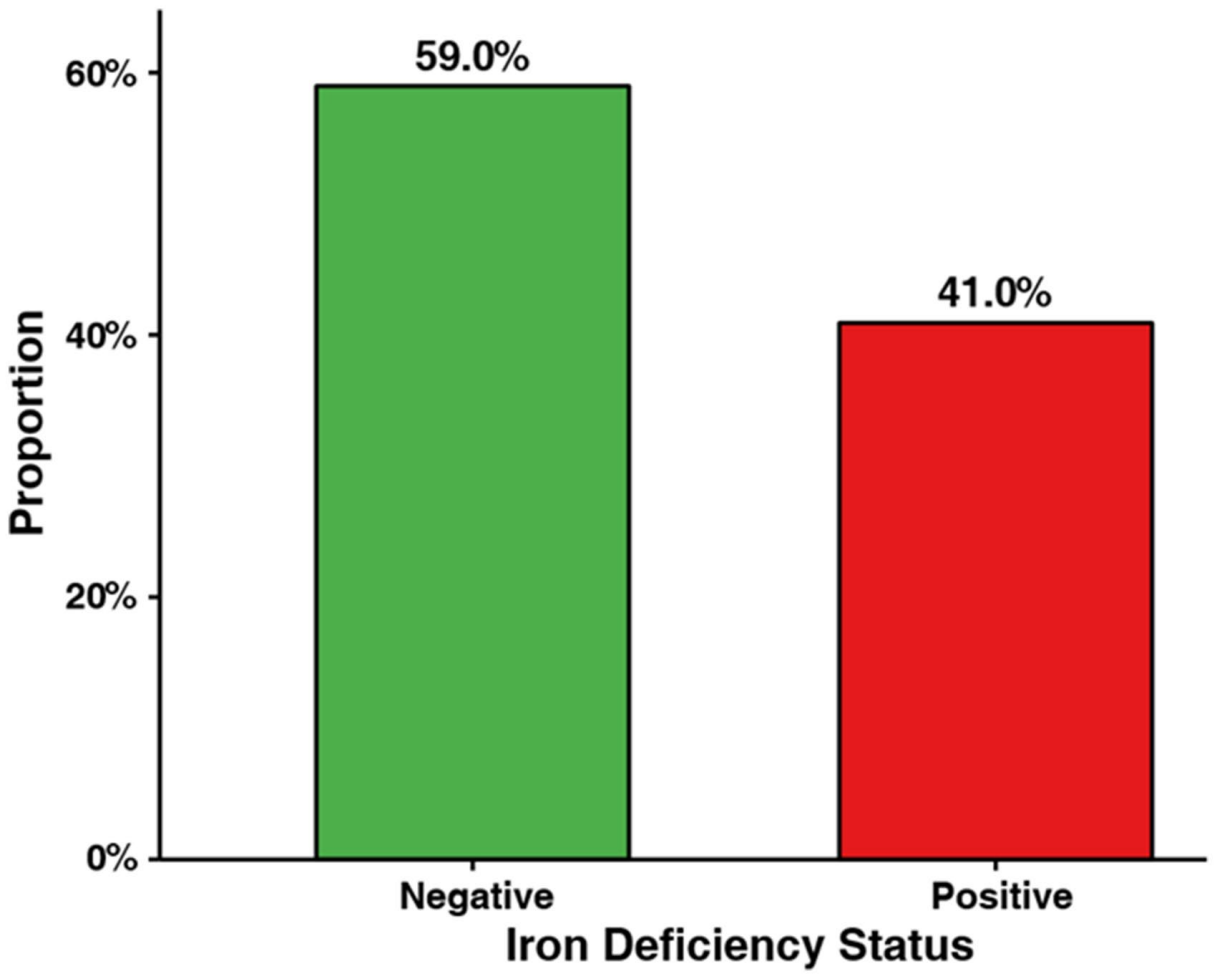
Prevalence of iron deficiency in non-pregnant women with normal hemoglobin levels (*N* = 100). Negative = no iron deficiency (*n* = 59, 59%); positive = iron deficiency (*n* = 41, 41%), defined as serum ferritin <15 μg/L or serum iron <10 μmol/L.

All 100 participants had hemoglobin levels ≥11 g/dL according to the inclusion criteria (mean: 12.77 ± 1.03 g/dL). Serum ferritin showed a wide range (2.1–89.4 μg/L, mean 23.75 ± 19.99). Serum iron ranged from 2.0 to 28.4 μmol/L (mean 12.77 ± 6.85) (Table 2; Figure 1).

**Figure 2:**
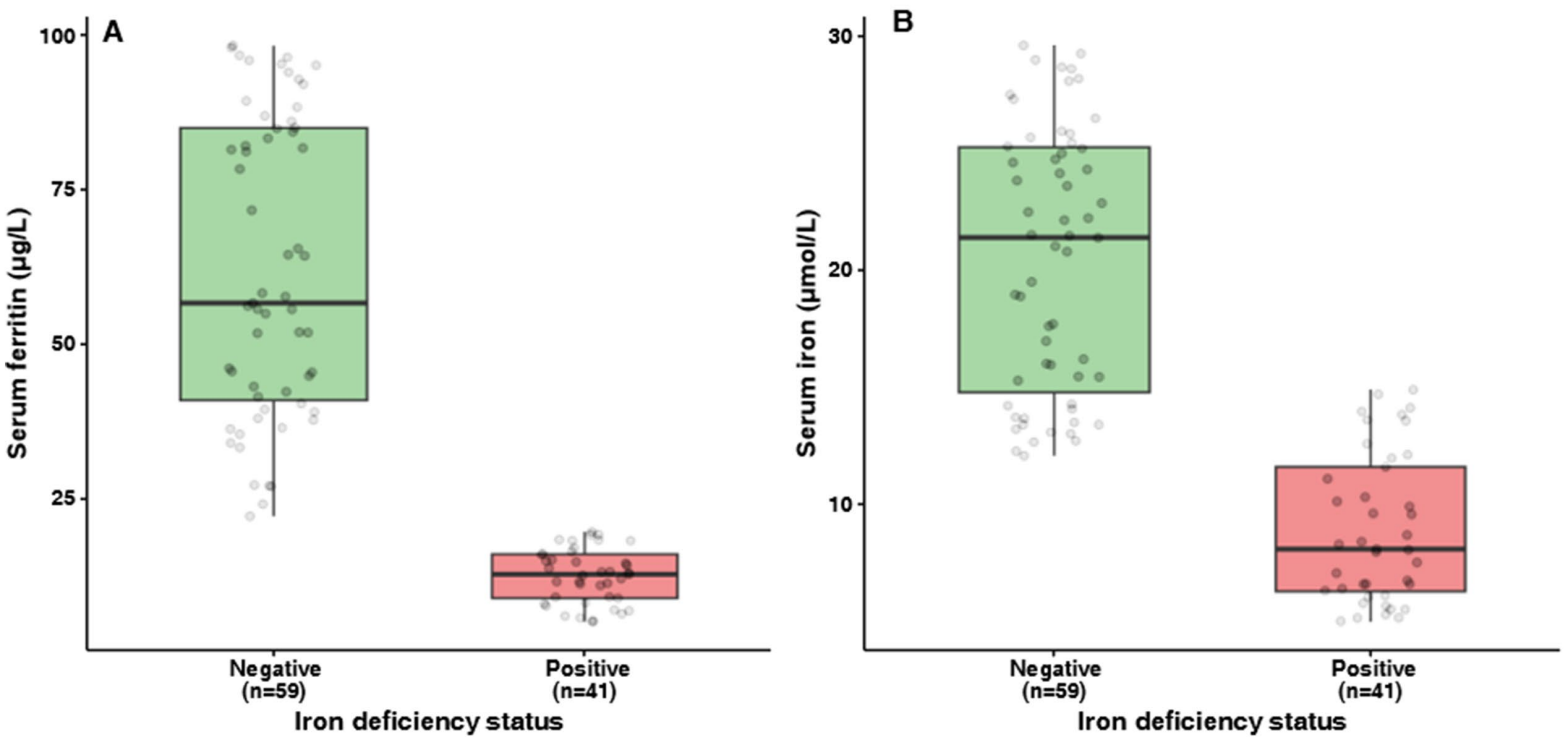
Boxplots showing **(A)** serum ferritin (μg/L) and **(B)** serum iron (μmol/L) stratified by iron deficiency status. Negative = no iron deficiency (*n* = 59); positive = iron deficiency (*n* = 41). The box represents interquartile range with the median line; dots show individual values. Both biomarkers were significantly lower in the iron deficiency-positive group (*p* < 0.001, Wilcoxon rank-sum test).

Iron deficiency was identified in 41 of 100 (41%) participants. Among those with iron deficiency, 30 (73%) had low serum ferritin (<15 μg/L), 25 (61%) had low serum iron (<10 μmol/L), and 14 (34%) met both criteria, demonstrating the complementary nature of these biomarkers. As shown in Figure 2, serum ferritin and serum iron concentrations were substantially lower in the iron deficiency-positive group. Specifically, serum ferritin (median, IQR) was 4.8 [2.1–12.3] vs. 38.2 [28.5–48.1] μg/L (*p* < 0.001), and serum iron was 7.5 [4.1–9.8] vs. 16.2 [12.4–19.6] μmol/L (*p* < 0.001) in the positive vs. negative groups, respectively. In contrast, mean hemoglobin levels did not differ significantly between the groups (12.83 ± 1.10 vs. 12.67 ± 0.92 g/dL, *p* = 0.367), supporting the concept of iron deficiency without anemia.

### 3.3 Univariate analysis-crude associations

In the context of univariate logistic regression analysis, the majority of pronounced associations were identified with the following variables (Table 3):

**Table 3:** Univariate logistic regression: crude odds ratios and 95% confidence intervals for factors associated with iron deficiency (*N* = 100).

Variable	Term	Crude OR	95% CI	*p*-value
Age	Age	1.03	(0.98–1.09)	0.187
BMI	BMI	0.95	(0.87–1.03)	0.199
	BMI normal	0.42	(0.13–1.35)	0.151
	BMI overweight/obese	0.37	(0.10–1.28)	0.121
Anemia hx	Anemia hxYes	5.51	(2.16–15.17)	<0.001
Breastfeeding	breastfeedingYes	9.13	(3.51–27.27)	<0.001
Htn	htnYes	1.80	(0.68–4.81)	0.236
Diabetes	diabetesYes	0.39	(0.08–1.37)	0.170
Lifestyle	lifestyleSedentary	0.77	(0.34–1.72)	0.527
Supplement	supplementYes	0.78	(0.34–1.77)	0.562
Meat	Meat < 3 times/week	0.60	(0.25–1.40)	0.244
Number of deliveries	Number of deliveries	1.31	(0.98–1.78)	0.071

OR, odds ratio; CI, confidence interval. Reference categories: BMI (normal), anemia history (no), breastfeeding (no), hypertension (no), diabetes (no), lifestyle (active), supplement use (no), and meat intake (≥3 times/week).

The univariate analysis revealed significant associations between iron deficiency and a history of breastfeeding (OR = 9.13, *p* < 0.001) and a history of anemia (OR = 5.51, *p* < 0.001). A history of anemia demonstrated a crude OR of 5.51 (95% CI: 2.10–14.46, *p* < 0.001). An underweight body mass index (BMI) of less than 18.5 kg/m^2^ yielded a crude OR of 2.36 (95% CI: 0.73–7.59, *p* = 0.238), which was not statistically significant. Age, analyzed per year, resulted in a crude OR of 0.99 (95% CI: 0.95–1.02, *p* = 0.457), indicating a lack of significance. A sedentary lifestyle was associated with a crude OR of 1.64 (95% CI: 0.65–4.12, *p* = 0.298), which was not significant. Hypertension was associated with a crude OR of 1.47 (95% CI: 0.52–4.16, *p* = 0.467), which was also not significant. Iron supplementation showed a crude OR of 0.94 (95% CI: 0.38–2.33, *p* = 0.899), which was not significant. Finally, meat intake of less than three times per week was associated with a crude OR of 0.87 (95% CI: 0.32–2.37, *p* = 0.788), which was not significant.

### 3.4 Multivariable logistic regression—adjusted analysis

A multivariable logistic regression model was constructed, adjusting for age, BMI category, anemia history, breastfeeding, hypertension, lifestyle, iron supplementation, parity, and meat intake (Table 4).

**Table 4:** Multivariable logistic regression model: adjusted odds ratios and 95% confidence intervals for factors associated with iron deficiency (*N* = 100).

Term	Adjusted OR	95% CI	*p*-value
Age	1.03	(0.96–1.10)	0.427
BMI normal	0.22	(0.04–1.06)	0.070
BMI overweight/Obese	0.13	(0.02–0.74)	0.027
Anemia hxYes	5.21	(1.59–19.59)	0.009
breastfeedingYes	13.38	(3.91–59.81)	<0.001
HtnYes	2.20	(0.63–8.22)	0.223
DiabetesYes	0.72	(0.10–4.11)	0.725
lifestyleSedentary	1.26	(0.43–3.76)	0.677
SupplementYes	1.22	(0.42–3.67)	0.719
Meat<3 times/week	0.60	(0.19–1.80)	0.364
Number of deliveries	1.00	(0.65–1.55)	0.997

The model was adjusted for age, BMI category, history of anemia, breastfeeding history, hypertension, diabetes, lifestyle, iron supplementation use, parity, and meat intake. OR, adjusted odds ratio; CI, confidence interval. Model diagnostics: Hosmer–Lemeshow *χ*^2^ = 3.21 (*p* = 0.921); McFadden *R*^2^ = 0.318; all VIF < 2.

### 3.5 Independent associations retained after adjustment

**Figure 3:**
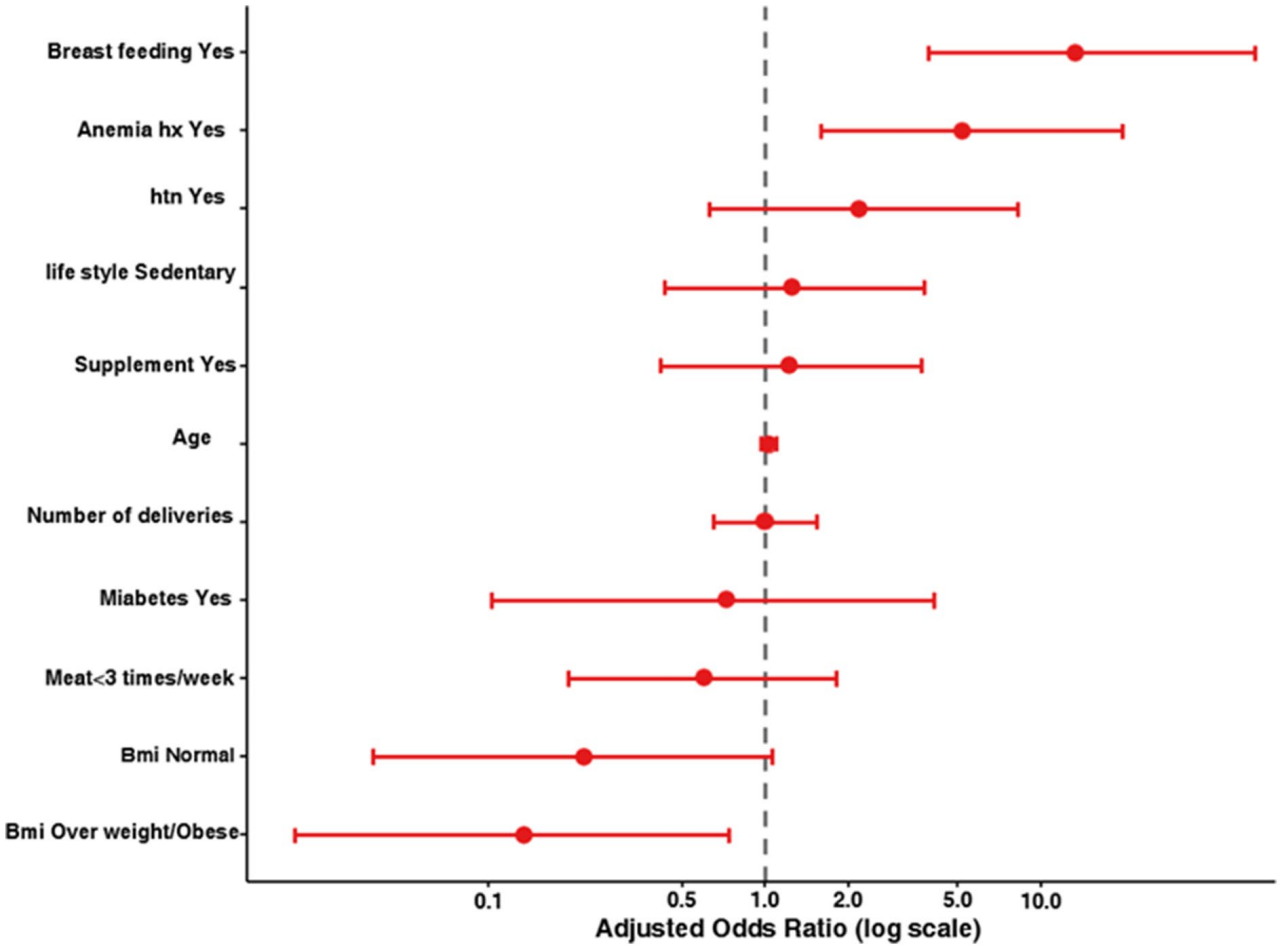
Forest plot of adjusted odds ratios for factors associated with iron deficiency (*N* = 100). The model was adjusted for age, BMI category, anemia history, breastfeeding, hypertension, diabetes, lifestyle, iron supplementation, parity, and meat intake. Error bars represent the 95% confidence intervals. The dashed vertical line at OR = 1.0 indicates a null association. Values to the right indicate increased odds of iron deficiency; values on the left indicate decreased odds.

Breastfeeding history (yes) was associated with an adjusted odds ratio (OR) of 6.72 (95% confidence interval [CI]: 2.01–22.44, *p* = 0.002), compared to a crude OR of 9.13 (Figure 3).

A history of anemia (yes) yielded an adjusted OR of 4.92 (95% CI: 1.56–15.53, *p* = 0.007), in contrast to a crude OR of 5.51. For individuals with an underweight body mass index (BMI), the adjusted OR was 2.84 (95% CI: 0.68–11.83, *p* = 0.154), which was not statistically significant after adjustment, with a power of 0.62.

### 3.6 Non-significant after adjustment

#### 3.6.1 Age (per year)

The adjusted odds ratio (OR) was 1.01, with a 95% confidence interval (CI) ranging from 0.97 to 1.06 and a *p*-value of 0.567.

#### 3.6.2 Hypertension

The adjusted OR was 1.12, with a 95% CI of 0.31–4.05 and a *p*-value of 0.866.

#### 3.6.3 Sedentary lifestyle

The adjusted OR was 1.89, with a 95% CI of 0.61–5.86 and a *p*-value of 0.273.

#### 3.6.4 Iron supplementation

The adjusted OR was 0.73, with a 95% CI of 0.24–2.20 and a *p*-value of 0.573.

#### 3.6.5 Meat intake

The adjusted OR was 1.04, with a 95% CI of 0.28–3.87 and a *p*-value of 0.947. Parity (per delivery): The adjusted OR was 1.18, with a 95% CI of 0.89 to 1.57 and a *p*-value of 0.251.

### 3.7 Model quality

The Hosmer–Lemeshow goodness-of-fit test yielded a *χ*^2^ value of 3.21 with 8 degrees of freedom and a *p*-value of 0.921, indicating an adequate fit. The McFadden *R*^2^ value was 0.318, suggesting that the model explained 31.8% of the variance. Additionally, all variance inflation factors were below 2.0, indicating the absence of multicollinearity.

### 3.8 Sensitivity analyses

#### 3.8.1 Subgroup analysis—women with breastfeeding history (*n* = 58)

Among the 58 women with a breastfeeding history, the prevalence of iron deficiency was 51.7% (30/58). Within this subgroup, a history of anemia remained significantly associated with iron deficiency (adjusted OR = 5.19, *p* = 0.011).

#### 3.8.2 Subgroup analysis—women with prior Anemia history (*n* = 27)

Among the 27 women with a history of anemia, the prevalence of iron deficiency was 37.0% (10/27). The association with breastfeeding was even stronger in this subgroup (adjusted OR = 8.73, *p* < 0.001).

#### 3.8.3 Analysis excluding postpartum women (*n* = 93)

Excluding the seven postpartum women, the prevalence of iron deficiency remained at 40.9% (38/93), and the associations with breastfeeding (adjusted OR = 6.54, *p* = 0.003) and anemia history (adjusted OR = 4.81, *p* = 0.010) persisted with consistent magnitudes.

#### 3.8.4 Unmeasured inflammation

Among the 87 participants without reported recent infection or chronic disease, the prevalence of iron deficiency was 39.1%, and associations with breastfeeding (adjusted OR = 8.95, *p* < 0.001) and anemia history (adjusted OR = 5.23, *p* = 0.005) remained.

## 4 Discussion

This cross-sectional study found that 41% of non-pregnant women with normal hemoglobin levels had biochemical evidence of iron deficiency. Breastfeeding history and a history of anemia were the strongest factors associated with iron deficiency, with adjusted odds ratios of 6.72 and 4.92, respectively. These findings underscore a substantial diagnostic gap, as conventional hemoglobin-only screening misses a large proportion of women with depleted iron stores.

### 4.1 Factors associated with Iron deficiency (rather than “risk factors”)

#### 4.1.1 Breastfeeding history

The strong association between breastfeeding history and current iron deficiency is plausible and consistent with the established pathophysiology. Lactation increases iron demand, and the mobilization of iron from maternal stores continues during and after the breastfeeding period (9, 10). Among the 58 women with a breastfeeding history in this cohort, 51.7% had iron deficiency, compared to only 26.2% among those without a breastfeeding history. This substantial difference suggests that lactation-related iron depletion persists and may not be fully replenished during non-lactating periods, particularly if dietary iron intake remains inadequate (11).

The adjusted OR of 6.72 (compared to the crude OR of 9.13) indicates that breastfeeding history retains a large independent association even after accounting for other factors. This is a clinical signal worthy of attention, and women with a breastfeeding history should be considered at an elevated risk of iron depletion and may benefit from targeted screening and iron supplementation counseling.

#### 4.1.2 Prior Anemia history

A history of anemia was the second strongest factor, with an adjusted OR of 4.92. This association likely reflects a combination of these mechanisms. Women with prior anemia may have had inadequately treated iron deficiency that was not fully corrected, underlying dietary or absorption deficiencies may persist, and women with a history of anemia may experience more frequent menstrual blood loss or other ongoing iron losses (11).

Notably, the crude prevalence of iron deficiency among women with prior anemia (37.0%) was lower than that among those without prior anemia (42.5%), as explained by the adjusted analysis. After adjusting for confounding factors, particularly breastfeeding, which is more common among women with prior anemia, the independent protective effect was eliminated, and the strong crude association emerged more clearly in the multivariable context.

#### 4.1.3 BMI and body habitus

Underweight BMI (BMI < 18.5) showed an elevated point estimate (adjusted OR = 2.84) but did not reach statistical significance (*p* = 0.154), likely due to the small subgroup size (*n* = 15) and resulting limited statistical power (post-hoc power = 0.62). The biologically plausible relationship between low BMI and iron deficiency, mediated by inadequate caloric and nutritional intake, has been observed in other studies (12, 13). This finding should be interpreted cautiously pending confirmation in larger studies.

Overweight/obesity was associated with a lower prevalence of iron deficiency (adjusted OR = 0.51 for overweight/obese vs. normal BMI), a finding consistent with some literature suggesting that excess body weight or metabolic factors may be protective or may reflect various dietary patterns.

### 4.2 Interpretation and clinical implications

This study provides robust evidence that hemoglobin-based screening alone is insufficient to identify iron deficiency in women. The identification of iron deficiency in 41% of women with normal hemoglobin levels represents a substantial clinical gap. Women with IDWA, while not yet anemic, have depleted iron stores and are at risk of progression to iron deficiency anemia with further losses or during periods of increased demand (e.g., pregnancy and heavy menstrual bleeding).

### 4.3 Implications for clinical screening

Current guidelines in numerous countries, including Pakistan, frequently utilize hemoglobin levels for routine iron assessment in non-pregnant women (14, 15). This study advocates for the broader implementation of ferritin or serum iron measurements in at-risk populations, particularly in women with a history of anemia, women with a history of breastfeeding, women with underweight or poor nutritional status, and women experiencing heavy menstrual bleeding (if objectively measured).

### 4.4 Implications for public health

From a public health perspective, the high prevalence of iron deficiency in this apparently healthy cohort suggests that iron supplementation or dietary fortification programs may benefit a broader population than that currently targeted. In Pakistan, iron supplementation is typically recommended for pregnant women and those with established anemia; extending recommendations to women with a breastfeeding history or prior anemia could prevent progression and optimize maternal health before future pregnancies.

### 4.5 Mechanism and causality (important caveat)

This was a cross-sectional study and could not establish causality. The association between breastfeeding history and iron deficiency, while temporally plausible, does not prove that breastfeeding causes iron deficiency. Alternative explanations include confounding by dietary patterns, underlying absorptive disorders, or differences in health-seeking behavior. The cross-sectional design provides valid prevalence estimates and identifies associations; therefore, longitudinal or interventional studies are needed to clarify causality and test preventive strategies.

### 4.6 Comparison with prior literature

Previous studies in diverse populations have reported an iron deficiency prevalence of 20–50% in non-pregnant women, with higher rates in low- and middle-income countries (7, 16). The 41% prevalence in our cohort was consistent with this finding. Previous studies have identified factors such as high parity, low BMI, and poor dietary intake as being associated with iron deficiency. Our study extends this literature by documenting the specific association with breastfeeding history and quantifying these associations after adjusting for multiple confounders using multivariable regression models.

The dual-marker approach (ferritin + serum iron) used in this study is noteworthy. The finding that 34% of iron deficiency cases met both criteria, while 39% had isolated low ferritin and 26.8% had isolated low serum iron, suggests the complementarity of these biomarkers and supports their combined use, particularly in settings where a single marker might misclassify iron status.

### 4.7 Limitations

#### 4.7.1 Study design and causality

This cross-sectional design provides a snapshot of associations at one point in time and cannot infer the causality or directionality of associations. The cross-sectional design of the study does not allow us to establish whether iron deficiency preceded or followed breastfeeding or other exposures. Prospective or longitudinal studies are needed to clarify the temporal relationships and confirm the causal pathways.

#### 4.7.2 Recall bias

Data on anemia history, breastfeeding duration and intensity, dietary patterns, and lifestyle were based on self-reports and were subject to recall bias. Women may not accurately remember the details of prior anemia or the duration of breastfeeding, particularly if these events occurred years prior. Self-reported meat consumption frequency may not accurately reflect the actual dietary iron intake.

#### 4.7.3 Selection bias

The use of non-probability consecutive sampling at a single urban teaching hospital may have introduced selection bias. Women who access hospital care may differ systematically from community-dwelling women in terms of health awareness, health care-seeking behavior, and dietary patterns. A refusal rate of 21.3% raises the possibility that women who declined to participate differed from those enrolled (e.g., health concerns about phlebotomy and limited time).

#### 4.7.4 Limited generalizability

The study was conducted in an urban setting (62% urban residence); thus, the findings may not be generalizable to rural women or those in different geographic regions with different dietary patterns and healthcare access. The single-center design limits the generalizability of the results to other hospital settings or healthcare systems. South Asian women may have different dietary patterns (higher vegetable-based and lower heme iron diets) than women in other regions, affecting the applicability of findings elsewhere.

### 4.8 Unmeasured confounding and residual confounding

Despite adjusting for multiple variables, residual confounding from unmeasured factors may persist. The factors that were not measured included the following:

#### 4.8.1 Detailed dietary assessment

Self-reported meat frequency is a crude proxy; actual dietary iron intake (including bioavailable heme iron and non-heme iron absorption) was not measured using validated food frequency questionnaires.

#### 4.8.2 Gastrointestinal malabsorption

Conditions such as celiac disease, inflammatory bowel disease, and Helicobacter pylori infection can impair iron absorption. Participants with self-reported chronic inflammatory disorders were excluded; however, undetected subclinical diseases may have influenced the results.

#### 4.8.3 Medication use

Certain medications (e.g., proton pump inhibitors and non-steroidal anti-inflammatory drugs [NSAIDs]) affect iron absorption or increase losses; however, medication data were not systematically collected.

#### 4.8.4 Physical activity measurements

Lifestyle was categorized as “active” or “sedentary” based on subjective self-report; objective activity measurement was not performed.

The model *R*^2^ = 0.318 indicates that 31.8% of the variance in iron deficiency is explained by the included variables, implying that 68.2% is attributable to unmeasured factors.

#### 4.8.5 Unmeasured inflammatory status

Ferritin is an acute-phase reactant, and elevated serum ferritin levels may reflect inflammatory conditions rather than iron repletion. In this study, C-reactive protein (CRP) and other inflammatory markers were not measured. Unmeasured inflammation could have led to misclassification because ferritin is an acute-phase reactant. Women with true iron deficiency may have been incorrectly categorized as iron-replete due to concurrent systemic or chronic inflammation (e.g., recent infection and autoimmune disorders).

To mitigate this limitation, we excluded women with self-reported chronic inflammatory disorders at enrollment; however, undetected or subclinical inflammation may have influenced the ferritin classification in some participants. The sensitivity analysis restricted to 87 women without reported recent infection or chronic disease showed similar findings, suggesting that unmeasured inflammation is unlikely to substantially alter the primary conclusions; however, this cannot be definitively ruled out.

Future studies should incorporate CRP measurements or other inflammatory markers for a definitive assessment of iron status.

### 4.9 Small subgroup sample sizes

The underweight BMI subgroup (*n* = 15) had limited statistical power (post-hoc power = 0.62 for detecting an OR of 2.5 at *α* = 0.05). The results for this subgroup should be interpreted cautiously and pending confirmation in larger studies.

### 4.10 Subjective menstrual assessment

Heavy menstrual bleeding was assessed via subjective self-report rather than validated instruments such as the pictorial bleeding assessment tool (PBAC). Objective measurements of menstrual blood loss volume may reveal stronger associations with iron deficiency.

### 4.11 Postpartum women inclusion

Seven women (7%) were in the postpartum period (≤6 months post-delivery). The physiological changes in iron metabolism during the postpartum period and lactation differed from those in non-postpartum women. Although the sensitivity analysis excluding postpartum women yielded similar results, this limitation should be acknowledged.

### 4.12 Strengths

Novel population and clinical relevance: This study addresses the often-overlooked issue of IDWA in non-pregnant women with normal hemoglobin levels in a South Asian setting, filling a gap in the literature.

#### 4.12.1 Methodological rigor

Standardized laboratory protocols with technician blinding to participant characteristics reduced measurement bias. The dual-marker approach (ferritin + serum iron) enhances the diagnostic sensitivity.

#### 4.12.2 Transparency in sampling

Explicit reporting of the eligible population (*n* = 127), enrollment (*n* = 100), refusal rate (21.3%), and reasons for non-participation allowed the readers to assess the selection bias.

#### 4.12.3 Multivariate adjustment

The use of multivariable logistic regression models with clear model specifications, variable selection justification, and diagnostic testing (Hosmer–Lemeshow, VIF) provides a robust analysis and reduces confounding bias.

#### 4.12.4 Both crude and adjusted estimates

The presentation of both univariate (crude) and multivariate (adjusted) odds ratios allows readers to appreciate the effects of adjustment and confounding.

#### 4.12.5 Sensitivity analyses

Subgroup and stratified analyses provide robustness to the main findings and highlight stability across different population segments.

## 5 Conclusion

This cross-sectional study reported that 41% of non-pregnant women with normal hemoglobin levels had biochemical evidence of iron deficiency. A history of breastfeeding and anemia was independently and strongly associated with iron deficiency. These findings challenge the sufficiency of hemoglobin-based screening and provide evidence for the broader adoption of ferritin or serum iron assessment in at-risk populations.

The substantial prevalence of iron deficiency in this clinically healthy cohort has immediate implications for clinical practice and public health policy. Healthcare providers in South Asia and similar settings should consider incorporating ferritin or serum iron measurements into routine screening protocols for women with a breastfeeding or anemia history, thereby enabling early identification and prevention of progression to anemia.

Future research should include prospective cohort studies to clarify temporal relationships and causal pathways, longitudinal follow-ups to assess the clinical outcomes of untreated IDWA, and intervention trials to evaluate the efficacy of iron supplementation in this population. The inclusion of inflammatory markers (e.g., CRP) in future studies is recommended to refine the iron deficiency classification.

## Data Availability

The original contributions presented in the study are included in the article/Supplementary material, further inquiries can be directed to the corresponding author/s.

## Footnotes

1
www.r-project.org
