# Correction: Iron deficiency in non-pregnant women with normal hemoglobin: a cross-sectional analysis of risk factors and clinical implications

**DOI:** 10.3389/fmed.2026.1794710

**Published:** 2026-02-02

**Authors:** Frontiers Production Office

**Affiliations:** Frontiers Media SA, Lausanne, Switzerland

**Keywords:** associations, breastfeeding, hemoglobin, iron deficiency without anemia, non-pregnant women, reproductive health, serum ferritin

An incorrect number was provided for Princess Nourah bint Abdulrahman University Researchers Supporting Project. The correct number is PNURSP2026R420.

The original version of this article has been updated.

