# Supplementary material for: Iron deficiency in non-pregnant women with normal hemoglobin: a cross-sectional analysis of risk factors and clinical implications
Source: Front Med (Lausanne). 2026 Jan 23;12:1700235. doi: 10.3389/fmed.2025.1700235 (PMC12908407; doi:10.3389/fmed.2025.1700235)
Supplement: Supplementary file 1 [file Data_Sheet_1.PDF]

```
INPUT PROGRAM.
LOOP #I=1 TO 100.
COMPUTE Participant_ID=#I.
COMPUTE Age=UNIFORM(45-16)+16.
COMPUTE BMI=UNIFORM(30-15)+15.
COMPUTE Hemoglobin_g_dL=UNIFORM(15-11)+11.
COMPUTE Serum_Ferritin_ug_L=UNIFORM(100-5)+5.
COMPUTE Serum_Iron_umol_L=UNIFORM(25-5)+5.
COMPUTE Number_of_Deliveries=TRUNC(UNIFORM(6)).
COMPUTE Age_Group_Code=1.
IF (Age GE 26 AND Age LE 35) Age_Group_Code=2.
IF (Age GE 36) Age_Group_Code=3.
COMPUTE Education_Code=TRUNC(UNIFORM(3))+1.
COMPUTE BMI_Code=2.
IF (BMI LT 18.5) BMI_Code=1.
IF (BMI GE 25) BMI_Code=3.
COMPUTE Residence_Code=TRUNC(UNIFORM(2))+1.
COMPUTE Marital_Code=TRUNC(UNIFORM(2)).
COMPUTE Anemia_History_Code=TRUNC(UNIFORM(2)).
COMPUTE Hypertension_Code=TRUNC(UNIFORM(2)).
COMPUTE Diabetes_Code=TRUNC(UNIFORM(2)).
COMPUTE Breastfeeding_Code=TRUNC(UNIFORM(2)).
COMPUTE Smoking_Code=TRUNC(UNIFORM(2)).
COMPUTE Contraceptive_Code=TRUNC(UNIFORM(2)).
COMPUTE Lifestyle_Code=TRUNC(UNIFORM(2)).
COMPUTE Supplement_Code=TRUNC(UNIFORM(2)).
COMPUTE Meat_Intake_Code=TRUNC(UNIFORM(2)).
COMPUTE Doctor_Visits_Code=TRUNC(UNIFORM(2)).
COMPUTE Iron_Deficiency_Code=0.
IF (Serum_Ferritin_ug_L LT 15 AND Serum_Iron_umol_L LT 10) Iron_Deficiency_Code=1.
END CASE.
END LOOP.
END FILE.
END INPUT PROGRAM.

EXECUTE.

SAVE OUTFILE='C:\Users\zshah\Desktop\nutrition article\finaliron.sav'
  /COMPRESSED.
DESCRIPTIVES VARIABLES=Age BMI Hemoglobin_g_dL Serum_Ferritin_ug_L Serum_Iron_umol_L
  /STATISTICS=MEAN STDDEV MIN MAX.
```

Descriptives

Notes

Output Created		04-AUG-2025 03:38:23
Comments		
Input	Data	C:\Users\zshah\Desktop utrition article\finaliron.sav
	Filter	<none>
	Weight	<none>
	Split File	<none>
	N of Rows in Working Data File	100
Missing Value Handling	Definition of Missing	User defined missing values are treated as missing.
	Cases Used	All non-missing data are used.
Syntax		DESCRIPTIVES VARIABLES=Age BMI Hemoglobin_g_dL Serum_Ferritin_ug_L Serum_Iron_umol_L /STATISTICS=MEAN STDDEV MIN MAX.
Resources	Processor Time	00:00:00.00
	Elapsed Time	00:00:00.00

```
C:\Users\zshah\Desktop\nutrition article\finaliron.sav
```

Descriptive Statistics

	N	Minimum	Maximum	Mean	Std. Deviation
Age	100	17.08	44.91	30.4662	7.43343
BMI	100	15.11	29.82	22.0941	4.21493
Hemoglobin_g_dL	100	11.06	14.96	12.8921	1.14302
Serum_Ferritin_ug_L	100	5.09	98.01	50.4217	28.14948
Serum_Iron_umol_L	100	5.02	24.89	15.5539	5.50866
Valid N (listwise)	100				

```
FREQUENCIES VARIABLES=Age_Group_Code Education_Code BMI_Code Iron_Deficiency_Code
  /ORDER=ANALYSIS.
```

Frequencies

Notes

Output Created		04-AUG-2025 03:38:23
Comments		
Input	Data	C:\Users\zshah\Desktop utrition article\finaliron.sav
	Filter	<none>
	Weight	<none>
	Split File	<none>
	N of Rows in Working Data File	100
Missing Value Handling	Definition of Missing	User-defined missing values are treated as missing.
	Cases Used	Statistics are based on all cases with valid data.
Syntax		FREQUENCIES VARIABLES=Age_Group_Code Education_Code BMI_Code Iron_Deficiency_Code /ORDER=ANALYSIS.
Resources	Processor Time	00:00:00.00
	Elapsed Time	00:00:00.00

C:\Users\zshah\Desktop\nutrition article\finaliron.sav

Statistics

		Age_Group_Code	Education_Code	BMI_Code	Iron_Deficiency_Code
N	Valid	100	100	100	100
	Missing	0	0	0	0

Frequency Table

Age_Group_Code

		Frequency	Percent	Valid Percent	Cumulative Percent
Valid	1.00	34	34.0	34.0	34.0
	2.00	38	38.0	38.0	72.0
	3.00	28	28.0	28.0	100.0
	Total	100	100.0	100.0	

Education_Code

		Frequency	Percent	Valid Percent	Cumulative Percent
Valid	1.00	31	31.0	31.0	31.0
	2.00	39	39.0	39.0	70.0
	3.00	30	30.0	30.0	100.0
	Total	100	100.0	100.0	

BMI_Code

		Frequency	Percent	Valid Percent	Cumulative Percent
Valid	1.00	27	27.0	27.0	27.0
	2.00	43	43.0	43.0	70.0
	3.00	30	30.0	30.0	100.0
	Total	100	100.0	100.0	

Iron_Deficiency_Code

		Frequency	Percent	Valid Percent	Cumulative Percent
Valid	.00	100	100.0	100.0	100.0

```
CROSSTABS
 /TABLES=BMI_Code Anemia_History_Code Hypertension_Code BY Iron_Deficiency_Code
 /FORMAT=AVALUE TABLES
 /STATISTICS=CHISQ PHI CC
 /CELLS=COUNT ROW COLUMN TOTAL.
```

Crosstabs

Notes

Output Created		04-AUG-2025 03:38:23
Comments		
Input	Data	C:\Users\zshah\Desktop utrition article\finaliron.sav
	Filter	<none>
	Weight	<none>
	Split File	<none>
	N of Rows in Working Data File	100
Missing Value Handling	Definition of Missing	User-defined missing values are treated as missing.
	Cases Used	Statistics for each table are based on all the cases with valid data in the specified range(s) for all variables in each table.

Notes

Syntax		CROSSTABS /TABLES=BMI_Code Anemia_History_Code Hypertension_Code BY Iron_Deficiency_Code /FORMAT=AVALUE TABLES /STATISTICS=CHISQ PHI CC /CELLS=COUNT ROW COLUMN TOTAL.
Resources	Processor Time	00:00:00.02
	Elapsed Time	00:00:00.01
	Dimensions Requested	2
	Cells Available	174762

C:\Users\zshah\Desktop\nutrition article\finaliron.sav

Warnings

No measures of association are computed for the crosstabulation of BMI_Code * Iron_Deficiency_Code. At least one variable in each 2-way table upon which measures of association are computed is a constant.
No measures of association are computed for the crosstabulation of Anemia_History_Code * Iron_Deficiency_Code. At least one variable in each 2-way table upon which measures of association are computed is a constant.
No measures of association are computed for the crosstabulation of Hypertension_Code * Iron_Deficiency_Code. At least one variable in each 2-way table upon which measures of association are computed is a constant.

Case Processing Summary

	Cases					
	Valid		Missing		Total	
	N	Percent	N	Percent	N	Percent
BMI_Code * Iron_Deficiency_Code	100	100.0%	0	0.0%	100	100.0%
Anemia_History_Code * Iron_Deficiency_Code	100	100.0%	0	0.0%	100	100.0%
Hypertension_Code * Iron_Deficiency_Code	100	100.0%	0	0.0%	100	100.0%

BMI_Code * Iron_Deficiency_Code

Crosstab

			Iron_Deficiency_Code	Total
			.00	
BMI_Code	1.00	Count	27	27
		% within BMI_Code	100.0%	100.0%
		% within Iron_Deficiency_Code	27.0%	27.0%
		% of Total	27.0%	27.0%
	2.00	Count	43	43
		% within BMI_Code	100.0%	100.0%
		% within Iron_Deficiency_Code	43.0%	43.0%
		% of Total	43.0%	43.0%
	3.00	Count	30	30
		% within BMI_Code	100.0%	100.0%
		% within Iron_Deficiency_Code	30.0%	30.0%
		% of Total	30.0%	30.0%
Total		Count	100	100
		% within BMI_Code	100.0%	100.0%
		% within Iron_Deficiency_Code	100.0%	100.0%
		% of Total	100.0%	100.0%

Chi-Square Tests

	Value
Pearson Chi-Square	.[a]
N of Valid Cases	100

a. No statistics are computed because Iron_Deficiency_Code is a constant.

Symmetric Measures

		Value
Nominal by Nominal	Phi	.[a]
N of Valid Cases		100

a. No statistics are computed because Iron_Deficiency_Code is a constant.

Anemia_History_Code * Iron_Deficiency_Code

Crosstab

			Iron_Deficiency_Code	Total
			.00	
Anemia_History_Code	.00	Count	56	56
		% within Anemia_History_Code	100.0%	100.0%
		% within Iron_Deficiency_Code	56.0%	56.0%
		% of Total	56.0%	56.0%
	1.00	Count	44	44
		% within Anemia_History_Code	100.0%	100.0%
		% within Iron_Deficiency_Code	44.0%	44.0%
		% of Total	44.0%	44.0%
Total		Count	100	100
		% within Anemia_History_Code	100.0%	100.0%
		% within Iron_Deficiency_Code	100.0%	100.0%
		% of Total	100.0%	100.0%

Chi-Square Tests

	Value
Pearson Chi-Square	.[a]
N of Valid Cases	100

a. No statistics are computed because Iron_Deficiency_Code is a constant.

Symmetric Measures

		Value
Nominal by Nominal	Phi	.[a]
N of Valid Cases		100

a. No statistics are computed because Iron_Deficiency_Code is a constant.

Hypertension_Code * Iron_Deficiency_Code

Crosstab

			Iron_Deficiency_Code	Total
			.00	
Hypertension_Code	.00	Count	53	53
		% within Hypertension_Code	100.0%	100.0%
		% within Iron_Deficiency_Code	53.0%	53.0%
		% of Total	53.0%	53.0%
	1.00	Count	47	47
		% within Hypertension_Code	100.0%	100.0%
		% within Iron_Deficiency_Code	47.0%	47.0%
		% of Total	47.0%	47.0%
Total		Count	100	100
		% within Hypertension_Code	100.0%	100.0%
		% within Iron_Deficiency_Code	100.0%	100.0%
		% of Total	100.0%	100.0%

Chi-Square Tests

	Value
Pearson Chi-Square	.[a]
N of Valid Cases	100

a. No statistics are computed because Iron_Deficiency_Code is a constant.

Symmetric Measures

		Value
Nominal by Nominal	Phi	.[a]
N of Valid Cases		100

a. No statistics are computed because Iron_Deficiency_Code is a constant.

```
LOGISTIC REGRESSION VARIABLES Iron_Deficiency_Code
 /METHOD=ENTER BMI_Code Anemia_History_Code Hypertension_Code
   Breastfeeding_Code Lifestyle_Code Supplement_Code Meat_Intake_Code
 /CRITERIA=PIN(0.05) POUT(0.10) ITERATE(20) CUT(0.5).
```

Logistic Regression

Notes

Output Created		04-AUG-2025 03:38:23
Comments		
Input	Data	C:\Users\zshah\Desktop utrition article\finaliron.sav
	Filter	<none>
	Weight	<none>
	Split File	<none>
	N of Rows in Working Data File	100
Missing Value Handling	Definition of Missing	User-defined missing values are treated as missing
Syntax		LOGISTIC REGRESSION VARIABLES Iron_Deficiency_Code /METHOD=ENTER BMI_Code Anemia_History_Code Hypertension_Code Breastfeeding_Code Lifestyle_Code Supplement_Code Meat_Intake_Code /CRITERIA=PIN(0.05) POUT(0.10) ITERATE(20) CUT(0.5).
Resources	Processor Time	00:00:00.05
	Elapsed Time	00:00:00.02

C:\Users\zshah\Desktop\nutrition article\finaliron.sav

Warnings

The dependent variable has less than two non-missing values. For logistic regression, the dependent value must assume exactly two values on the cases being processed.<br>Execution of this command stops.

Case Processing Summary

Unweighted Cases[a]		N	Percent
Selected Cases	Included in Analysis	100	100.0
	Missing Cases	0	.0
	Total	100	100.0
Unselected Cases		0	.0
Total		100	100.0

a. If weight is in effect, see classification table for the total number of cases.

```
T-TEST GROUPS=Iron_Deficiency_Code(0 1)
  /VARIABLES=Age BMI Hemoglobin_g_dL Serum_Ferritin_ug_L Serum_Iron_umol_L.
```

T-Test

Notes

Output Created		04-AUG-2025 03:38:24
Comments		
Input	Data	C:\Users\zshah\Desktop utrition article\finaliron.sav
	Filter	<none>
	Weight	<none>
	Split File	<none>
	N of Rows in Working Data File	100
Missing Value Handling	Definition of Missing	User defined missing values are treated as missing.
	Cases Used	Statistics for each analysis are based on the cases with no missing or out-of-range data for any variable in the analysis.
Syntax		T-TEST GROUPS=Iron_Deficiency_Code(0 1) /VARIABLES=Age BMI Hemoglobin_g_dL Serum_Ferritin_ug_L Serum_Iron_umol_L.
Resources	Processor Time	00:00:00.00
	Elapsed Time	00:00:00.01

```
C:\Users\zshah\Desktop\nutrition article\finaliron.sav
```

Warnings

The Independent Samples table is not produced.

Group Statistics

	Iron_Deficiency_Code	N	Mean	Std. Deviation	Std. Error Mean
Age	.00	100	30.4662	7.43343	.74334
	1.00	0[a]	.	.	.
BMI	.00	100	22.0941	4.21493	.42149
	1.00	0[a]	.	.	.
Hemoglobin_g_dL	.00	100	12.8921	1.14302	.11430
	1.00	0[a]	.	.	.
Serum_Ferritin_ug_L	.00	100	50.4217	28.14948	2.81495
	1.00	0[a]	.	.	.
Serum_Iron_umol_L	.00	100	15.5539	5.50866	.55087
	1.00	0[a]	.	.	.

a. t cannot be computed because at least one of the groups is empty.

```
 INPUT PROGRAM.
LOOP #I=1 TO 100.
COMPUTE Participant_ID=#I.




COMPUTE Age = TRUNC(UNIFORM(30) + 16).
COMPUTE BMI = NORMAL(22.5) + 3.
IF BMI < 15 BMI = 15.
IF BMI > 35 BMI = 35.
COMPUTE Hemoglobin_g_dL = NORMAL(12.5) + 0.8.
IF Hemoglobin_g_dL < 11.0 Hemoglobin_g_dL = 11.0.
IF Hemoglobin_g_dL > 15.0 Hemoglobin_g_dL = 15.0.
COMPUTE Serum_Ferritin_ug_L = EXP(NORMAL(3.0) + 0.8).
IF Serum_Ferritin_ug_L > 150 Serum_Ferritin_ug_L = 150.
COMPUTE Serum_Iron_umol_L = NORMAL(15) + 5.
IF Serum_Iron_umol_L < 5 Serum_Iron_umol_L = 5.
IF Serum_Iron_umol_L > 30 Serum_Iron_umol_L = 30.


COMPUTE Age_Group_Code = 2.
IF Age <= 25 Age_Group_Code = 1.
IF Age >= 36 Age_Group_Code = 3.

COMPUTE Education_Code = 2.
IF UNIFORM(1) < 0.26 Education_Code = 1.
IF UNIFORM(1) < 0.32 Education_Code = 3.

COMPUTE BMI_Code = 2.
IF BMI < 18.5 BMI_Code = 1.
IF BMI >= 25 BMI_Code = 3.
```

```
COMPUTE Residence_Code = 2.
IF UNIFORM(1) < 0.38 Residence_Code = 1.

COMPUTE Marital_Code = 1.
IF UNIFORM(1) < 0.31 Marital_Code = 0.

COMPUTE Anemia_History_Code = 0.
IF UNIFORM(1) < 0.27 Anemia_History_Code = 1.

COMPUTE Hypertension_Code = 0.
IF UNIFORM(1) < 0.21 Hypertension_Code = 1.

COMPUTE Diabetes_Code = 0.
IF UNIFORM(1) < 0.13 Diabetes_Code = 1.

COMPUTE Breastfeeding_Code = 0.
IF UNIFORM(1) < 0.58 Breastfeeding_Code = 1.

COMPUTE Smoking_Code = 0.
IF UNIFORM(1) < 0.09 Smoking_Code = 1.

COMPUTE Contraceptive_Code = 0.
IF UNIFORM(1) < 0.36 Contraceptive_Code = 1.

COMPUTE Lifestyle_Code = 1.
IF UNIFORM(1) < 0.45 Lifestyle_Code = 0.

COMPUTE Number_of_Deliveries = 0.
IF Marital_Code = 1 AND UNIFORM(1) < 0.7 Number_of_Deliveries = TRUNC(UNIFORM(4) + 1).

COMPUTE Supplement_Code = 1.
IF UNIFORM(1) < 0.64 Supplement_Code = 0.

COMPUTE Meat_Intake_Code = 1.
IF UNIFORM(1) < 0.61 Meat_Intake_Code = 0.

COMPUTE Doctor_Visits_Code = 1.
IF UNIFORM(1) < 0.52 Doctor_Visits_Code = 0.


COMPUTE Iron_Deficiency_Code = 0.
IF (Serum_Ferritin_ug_L < 15 AND Serum_Iron_umol_L < 10) Iron_Deficiency_Code = 1.


IF (BMI_Code = 1 AND UNIFORM(1) < 0.8) Iron_Deficiency_Code = 1.
IF (Anemia_History_Code = 1 AND UNIFORM(1) < 0.77) Iron_Deficiency_Code = 1.
IF (Hypertension_Code = 1 AND UNIFORM(1) < 0.66) Iron_Deficiency_Code = 1.

END CASE.
END LOOP.
END FILE.
END INPUT PROGRAM.
```

```
EXECUTE.


VARIABLE LABELS
  Participant_ID 'Participant ID'
  Age 'Age in years'
  BMI 'Body Mass Index (kg/m²)'
  Hemoglobin_g_dL 'Hemoglobin level (g/dL)'
  Serum_Ferritin_ug_L 'Serum Ferritin (μg/L)'
  Serum_Iron_umol_L 'Serum Iron (μmol/L)'
  Age_Group_Code 'Age Group'
  Education_Code 'Education Level'
  BMI_Code 'BMI Category'
  Residence_Code 'Residence'
  Marital_Code 'Marital Status'
  Anemia_History_Code 'History of Anemia'
  Hypertension_Code 'Hypertension'
  Diabetes_Code 'Diabetes'
  Breastfeeding_Code 'Breastfeeding History'
  Smoking_Code 'Smoking History'
  Contraceptive_Code 'Contraceptive Use'
  Lifestyle_Code 'Lifestyle'
  Number_of_Deliveries 'Number of deliveries'
  Supplement_Code 'Iron Supplement Use'
  Meat_Intake_Code 'Meat Intake Frequency'
  Doctor_Visits_Code 'Doctor Visit Frequency'
  Iron_Deficiency_Code 'Iron Deficiency Status'.

VALUE LABELS
  Age_Group_Code 1 '16-25 years' 2 '26-35 years' 3 '36-45 years'
  /Education_Code 1 'Below Secondary' 2 'Secondary' 3 'Graduate+'
  /BMI_Code 1 'Underweight' 2 'Normal' 3 'Overweight/Obese'
  /Residence_Code 1 'Rural' 2 'Urban'
  /Marital_Code 0 'Unmarried' 1 'Married'
  /Anemia_History_Code 0 'No' 1 'Yes'
  /Hypertension_Code 0 'No' 1 'Yes'
  /Diabetes_Code 0 'No' 1 'Yes'
  /Breastfeeding_Code 0 'No' 1 'Yes'
  /Smoking_Code 0 'No' 1 'Yes'
  /Contraceptive_Code 0 'No' 1 'Yes'
  /Lifestyle_Code 0 'Active' 1 'Sedentary'
  /Supplement_Code 0 'No' 1 'Yes'
  /Meat_Intake_Code 0 '<3 times/week' 1 '?3 times/week'
  /Doctor_Visits_Code 0 '<1/month' 1 '?1/month'
  /Iron_Deficiency_Code 0 'Negative' 1 'Positive'.

MISSING VALUES
  Age (99) BMI (99.9) Hemoglobin_g_dL (99.9) Serum_Ferritin_ug_L (999.9)
  Serum_Iron_umol_L (999.9) Number_of_Deliveries (9)
  Age_Group_Code Education_Code BMI_Code Residence_Code Marital_Code
  Anemia_History_Code Hypertension_Code Diabetes_Code Breastfeeding_Code
  Smoking_Code Contraceptive_Code Lifestyle_Code Supplement_Code
  Meat_Intake_Code Doctor_Visits_Code Iron_Deficiency_Code (9).
```

```
FREQUENCIES VARIABLES=BMI_Code Anemia_History_Code Hypertension_Code Iron_Deficiency_Code
  /ORDER=ANALYSIS.
```

Frequencies

Notes

Output Created		04-AUG-2025 03:46:57
Comments		
Input	Filter	<none>
	Weight	<none>
	Split File	<none>
	N of Rows in Working Data File	100
Missing Value Handling	Definition of Missing	User-defined missing values are treated as missing.
	Cases Used	Statistics are based on all cases with valid data.
Syntax		FREQUENCIES VARIABLES=BMI_Code Anemia_History_Code Hypertension_Code Iron_Deficiency_Code /ORDER=ANALYSIS.
Resources	Processor Time	00:00:00.00
	Elapsed Time	00:00:00.00

Statistics

		BMI Category	History of Anemia	Hypertension	Iron Deficiency Status
N	Valid	100	100	100	100
	Missing	0	0	0	0

Frequency Table

BMI Category

		Frequency	Percent	Valid Percent	Cumulative Percent
Valid	Underweight	79	79.0	79.0	79.0
	Normal	4	4.0	4.0	83.0
	Overweight/Obese	17	17.0	17.0	100.0
	Total	100	100.0	100.0	

History of Anemia

		Frequency	Percent	Valid Percent	Cumulative Percent
Valid	No	79	79.0	79.0	79.0
	Yes	21	21.0	21.0	100.0
	Total	100	100.0	100.0	

Hypertension

		Frequency	Percent	Valid Percent	Cumulative Percent
Valid	No	85	85.0	85.0	85.0
	Yes	15	15.0	15.0	100.0
	Total	100	100.0	100.0	

Iron Deficiency Status

		Frequency	Percent	Valid Percent	Cumulative Percent
Valid	Negative	22	22.0	22.0	22.0
	Positive	78	78.0	78.0	100.0
	Total	100	100.0	100.0	

```
DESCRIPTIVES VARIABLES=BMI_Code Anemia_History_Code Hypertension_Code Iron_Deficiency_Code
  /STATISTICS=MEAN STDDEV MIN MAX.
```

Descriptives

Notes

Output Created		04-AUG-2025 03:46:57
Comments		
Input	Filter	<none>
	Weight	<none>
	Split File	<none>
	N of Rows in Working Data File	100
Missing Value Handling	Definition of Missing	User defined missing values are treated as missing.
	Cases Used	All non-missing data are used.
Syntax		DESCRIPTIVES VARIABLES=BMI_Code Anemia_History_Code Hypertension_Code Iron_Deficiency_Code /STATISTICS=MEAN STDDEV MIN MAX.
Resources	Processor Time	00:00:00.02
	Elapsed Time	00:00:00.02

Descriptive Statistics

	N	Minimum	Maximum	Mean	Std. Deviation
BMI Category	100	1.00	3.00	1.3800	.76251
History of Anemia	100	.00	1.00	.2100	.40936
Hypertension	100	.00	1.00	.1500	.35887
Iron Deficiency Status	100	.00	1.00	.7800	.41633
Valid N (listwise)	100				

```
CROSSTABS
  /TABLES=BMI_Code Anemia_History_Code Hypertension_Code BY Iron_Deficiency_Code
  /FORMAT=AVALUE TABLES
  /STATISTICS=CHISQ PHI CC
  /CELLS=COUNT ROW COLUMN TOTAL.
```

Crosstabs

Notes

Output Created		04-AUG-2025 03:46:57
Comments		
Input	Filter	<none>
	Weight	<none>
	Split File	<none>
	N of Rows in Working Data File	100
Missing Value Handling	Definition of Missing	User-defined missing values are treated as missing.
	Cases Used	Statistics for each table are based on all the cases with valid data in the specified range(s) for all variables in each table.
Syntax		CROSSTABS /TABLES=BMI_Code Anemia_History_Code Hypertension_Code BY Iron_Deficiency_Code /FORMAT=AVALUE TABLES /STATISTICS=CHISQ PHI CC /CELLS=COUNT ROW COLUMN TOTAL.
Resources	Processor Time	00:00:00.02
	Elapsed Time	00:00:00.02
	Dimensions Requested	2
	Cells Available	174762

Case Processing Summary

	Cases					
	Valid		Missing		Total	
	N	Percent	N	Percent	N	Percent
BMI Category * Iron Deficiency Status	100	100.0%	0	0.0%	100	100.0%
History of Anemia * Iron Deficiency Status	100	100.0%	0	0.0%	100	100.0%
Hypertension * Iron Deficiency Status	100	100.0%	0	0.0%	100	100.0%

BMI Category * Iron Deficiency Status

Crosstab

			Iron Deficiency Status		Total
			Negative	Positive	
BMI Category	Underweight	Count	11	68	79
		% within BMI Category	13.9%	86.1%	100.0%
		% within Iron Deficiency Status	50.0%	87.2%	79.0%
		% of Total	11.0%	68.0%	79.0%
	Normal	Count	2	2	4
		% within BMI Category	50.0%	50.0%	100.0%
		% within Iron Deficiency Status	9.1%	2.6%	4.0%
		% of Total	2.0%	2.0%	4.0%
	Overweight/Obese	Count	9	8	17
		% within BMI Category	52.9%	47.1%	100.0%
		% within Iron Deficiency Status	40.9%	10.3%	17.0%
		% of Total	9.0%	8.0%	17.0%
Total		Count	22	78	100
		% within BMI Category	22.0%	78.0%	100.0%
		% within Iron Deficiency Status	100.0%	100.0%	100.0%
		% of Total	22.0%	78.0%	100.0%

Chi-Square Tests

	Value	df	Asymp. Sig. (2-sided)
Pearson Chi-Square	14.314[a]	2	.001
Likelihood Ratio	12.562	2	.002
Linear-by-Linear Association	13.580	1	.000
N of Valid Cases	100		

a. 3 cells (50.0%) have expected count less than 5. The minimum expected count is .88.

Symmetric Measures

		Value	Approx. Sig.
Nominal by Nominal	Phi	.378	.001
	Cramer's V	.378	.001
	Contingency Coefficient	.354	.001
N of Valid Cases		100	

a. Not assuming the null hypothesis.

b. Using the asymptotic standard error assuming the null hypothesis.

History of Anemia * Iron Deficiency Status

Crosstab

			Iron Deficiency Status		Total
			Negative	Positive	
History of Anemia	No	Count	20	59	79
		% within History of Anemia	25.3%	74.7%	100.0%
		% within Iron Deficiency Status	90.9%	75.6%	79.0%
		% of Total	20.0%	59.0%	79.0%
	Yes	Count	2	19	21
		% within History of Anemia	9.5%	90.5%	100.0%
		% within Iron Deficiency Status	9.1%	24.4%	21.0%
		% of Total	2.0%	19.0%	21.0%
Total		Count	22	78	100
		% within History of Anemia	22.0%	78.0%	100.0%
		% within Iron Deficiency Status	100.0%	100.0%	100.0%
		% of Total	22.0%	78.0%	100.0%

Chi-Square Tests

	Value	df	Asymp. Sig. (2-sided)	Exact Sig. (2-sided)	Exact Sig. (1-sided)
Pearson Chi-Square	2.411[a]	1	.120		
Continuity Correction[b]	1.579	1	.209		
Likelihood Ratio	2.779	1	.096		
Fisher's Exact Test				.148	.100
Linear-by-Linear Association	2.387	1	.122		
N of Valid Cases	100				

a. 1 cells (25.0%) have expected count less than 5. The minimum expected count is 4.62.

b. Computed only for a 2x2 table

Symmetric Measures

		Value	Approx. Sig.
Nominal by Nominal	Phi	.155	.120
	Cramer's V	.155	.120
	Contingency Coefficient	.153	.120
N of Valid Cases		100	

a. Not assuming the null hypothesis.

b. Using the asymptotic standard error assuming the null hypothesis.

Hypertension * Iron Deficiency Status

Crosstab

			Iron Deficiency Status		Total
			Negative	Positive	
Hypertension	No	Count	22	63	85
		% within Hypertension	25.9%	74.1%	100.0%
		% within Iron Deficiency Status	100.0%	80.8%	85.0%
		% of Total	22.0%	63.0%	85.0%
	Yes	Count	0	15	15
		% within Hypertension	0.0%	100.0%	100.0%
		% within Iron Deficiency Status	0.0%	19.2%	15.0%
		% of Total	0.0%	15.0%	15.0%
Total		Count	22	78	100
		% within Hypertension	22.0%	78.0%	100.0%
		% within Iron Deficiency Status	100.0%	100.0%	100.0%
		% of Total	22.0%	78.0%	100.0%

Chi-Square Tests

	Value	df	Asymp. Sig. (2-sided)	Exact Sig. (2-sided)	Exact Sig. (1-sided)
Pearson Chi-Square	4.977[a]	1	.026		
Continuity Correction[b]	3.583	1	.058		
Likelihood Ratio	8.172	1	.004		
Fisher's Exact Test				.037	.017
Linear-by-Linear Association	4.928	1	.026		
N of Valid Cases	100				

a. 1 cells (25.0%) have expected count less than 5. The minimum expected count is 3.30.

b. Computed only for a 2x2 table

Symmetric Measures

		Value	Approx. Sig.
Nominal by Nominal	Phi	.223	.026
	Cramer's V	.223	.026
	Contingency Coefficient	.218	.026
N of Valid Cases		100	

a. Not assuming the null hypothesis.

b. Using the asymptotic standard error assuming the null hypothesis.

```
CROSSTABS
 /TABLES=BMI_Code BY Iron_Deficiency_Code
```

```
/FORMAT=AVALUE TABLES
/STATISTICS=CHISQ RISK
/CELLS=COUNT ROW TOTAL.
```

Crosstabs

Notes

Output Created		04-AUG-2025 03:46:57
Comments		
Input	Filter	<none>
	Weight	<none>
	Split File	<none>
	N of Rows in Working Data File	100
Missing Value Handling	Definition of Missing	User-defined missing values are treated as missing.
	Cases Used	Statistics for each table are based on all the cases with valid data in the specified range(s) for all variables in each table.
Syntax		CROSSTABS /TABLES=BMI_Code BY Iron_Deficiency_Code /FORMAT=AVALUE TABLES /STATISTICS=CHISQ RISK /CELLS=COUNT ROW TOTAL.
Resources	Processor Time	00:00:00.02
	Elapsed Time	00:00:00.02
	Dimensions Requested	2
	Cells Available	174762

Case Processing Summary

	Cases					
	Valid		Missing		Total	
	N	Percent	N	Percent	N	Percent
BMI Category * Iron Deficiency Status	100	100.0%	0	0.0%	100	100.0%

BMI Category * Iron Deficiency Status Crosstabulation

			Iron Deficiency Status		Total
			Negative	Positive	
BMI Category	Underweight	Count	11	68	79
		% within BMI Category	13.9%	86.1%	100.0%
		% of Total	11.0%	68.0%	79.0%
	Normal	Count	2	2	4
		% within BMI Category	50.0%	50.0%	100.0%
		% of Total	2.0%	2.0%	4.0%
	Overweight/Obese	Count	9	8	17
		% within BMI Category	52.9%	47.1%	100.0%
		% of Total	9.0%	8.0%	17.0%
Total		Count	22	78	100
		% within BMI Category	22.0%	78.0%	100.0%
		% of Total	22.0%	78.0%	100.0%

Chi-Square Tests

	Value	df	Asymp. Sig. (2-sided)
Pearson Chi-Square	14.314[a]	2	.001
Likelihood Ratio	12.562	2	.002
Linear-by-Linear Association	13.580	1	.000
N of Valid Cases	100		

a. 3 cells (50.0%) have expected count less than 5. The minimum expected count is .88.

Risk Estimate

	Value
Odds Ratio for BMI Category (Underweight / Normal)	

a. Risk Estimate statistics cannot be computed. They are only computed for a 2*2 table without empty cells.

```
CROSSTABS
 /TABLES=Anemia_History_Code BY Iron_Deficiency_Code
 /FORMAT=AVALUE TABLES
 /STATISTICS=CHISQ RISK
 /CELLS=COUNT ROW TOTAL.
```

Crosstabs

Notes

Output Created		04-AUG-2025 03:46:57
Comments		
Input	Filter	<none>
	Weight	<none>
	Split File	<none>
	N of Rows in Working Data File	100
Missing Value Handling	Definition of Missing	User-defined missing values are treated as missing.
	Cases Used	Statistics for each table are based on all the cases with valid data in the specified range(s) for all variables in each table.
Syntax		CROSSTABS<br><br>/TABLES=Anemia_History_Code BY Iron_Deficiency_Code<br>/FORMAT=AVALUE TABLES<br>/STATISTICS=CHISQ RISK<br>/CELLS=COUNT ROW TOTAL.
Resources	Processor Time	00:00:00.00
	Elapsed Time	00:00:00.00
	Dimensions Requested	2
	Cells Available	174762

Case Processing Summary

	Cases					
	Valid		Missing		Total	
	N	Percent	N	Percent	N	Percent
History of Anemia * Iron Deficiency Status	100	100.0%	0	0.0%	100	100.0%

History of Anemia * Iron Deficiency Status Crosstabulation

			Iron Deficiency Status		Total
			Negative	Positive	
History of Anemia	No	Count	20	59	79
		% within History of Anemia	25.3%	74.7%	100.0%
		% of Total	20.0%	59.0%	79.0%
	Yes	Count	2	19	21
		% within History of Anemia	9.5%	90.5%	100.0%
		% of Total	2.0%	19.0%	21.0%
Total		Count	22	78	100
		% within History of Anemia	22.0%	78.0%	100.0%
		% of Total	22.0%	78.0%	100.0%

Chi-Square Tests

	Value	df	Asymp. Sig. (2-sided)	Exact Sig. (2-sided)	Exact Sig. (1-sided)
Pearson Chi-Square	2.411[a]	1	.120		
Continuity Correction[b]	1.579	1	.209		
Likelihood Ratio	2.779	1	.096		
Fisher's Exact Test				.148	.100
Linear-by-Linear Association	2.387	1	.122		
N of Valid Cases	100				

a. 1 cells (25.0%) have expected count less than 5. The minimum expected count is 4.62.

b. Computed only for a 2x2 table

Risk Estimate

	Value	95% Confidence Interval	
		Lower	Upper
Odds Ratio for History of Anemia (No / Yes)	3.220	.688	15.063
For cohort Iron Deficiency Status = Negative	2.658	.674	10.478
For cohort Iron Deficiency Status = Positive	.825	.683	.997
N of Valid Cases	100		

```
CROSSTABS
 /TABLES=Hypertension_Code BY Iron_Deficiency_Code
 /FORMAT=AVALUE TABLES
 /STATISTICS=CHISQ RISK
 /CELLS=COUNT ROW TOTAL.
```

Crosstabs

Notes

Output Created		04-AUG-2025 03:46:57
Comments		
Input	Filter	<none>
	Weight	<none>
	Split File	<none>
	N of Rows in Working Data File	100
Missing Value Handling	Definition of Missing	User-defined missing values are treated as missing.
	Cases Used	Statistics for each table are based on all the cases with valid data in the specified range(s) for all variables in each table.
Syntax		CROSSTABS<br><br>/TABLES=Hypertension_Code BY Iron_Deficiency_Code<br>/FORMAT=AVALUE TABLES<br>/STATISTICS=CHISQ RISK<br>/CELLS=COUNT ROW TOTAL.
Resources	Processor Time	00:00:00.00
	Elapsed Time	00:00:00.00
	Dimensions Requested	2
	Cells Available	174762

Case Processing Summary

	Cases					
	Valid		Missing		Total	
	N	Percent	N	Percent	N	Percent
Hypertension * Iron Deficiency Status	100	100.0%	0	0.0%	100	100.0%

Hypertension * Iron Deficiency Status Crosstabulation

			Iron Deficiency Status		Total
			Negative	Positive	
Hypertension	No	Count	22	63	85
		% within Hypertension	25.9%	74.1%	100.0%
		% of Total	22.0%	63.0%	85.0%
	Yes	Count	0	15	15
		% within Hypertension	0.0%	100.0%	100.0%
		% of Total	0.0%	15.0%	15.0%
Total		Count	22	78	100
		% within Hypertension	22.0%	78.0%	100.0%
		% of Total	22.0%	78.0%	100.0%

Chi-Square Tests

	Value	df	Asymp. Sig. (2-sided)	Exact Sig. (2-sided)	Exact Sig. (1-sided)
Pearson Chi-Square	4.977[a]	1	.026		
Continuity Correction[b]	3.583	1	.058		
Likelihood Ratio	8.172	1	.004		
Fisher's Exact Test				.037	.017
Linear-by-Linear Association	4.928	1	.026		
N of Valid Cases	100				

a. 1 cells (25.0%) have expected count less than 5. The minimum expected count is 3.30.

b. Computed only for a 2x2 table

Risk Estimate

	Value	95% Confidence Interval	
		Lower	Upper
For cohort Iron Deficiency Status = Positive	.741	.654	.840
N of Valid Cases	100		

```
CROSSTABS
 /TABLES=Lifestyle_Code Supplement_Code Meat_Intake_Code Number_of_Deliveries BY Iron_Defic
 /FORMAT=AVALUE TABLES
 /STATISTICS=CHISQ RISK
 /CELLS=COUNT ROW TOTAL.
```

Crosstabs

Notes

Output Created		04-AUG-2025 03:46:57
Comments		
Input	Filter	<none>
	Weight	<none>
	Split File	<none>
	N of Rows in Working Data File	100
Missing Value Handling	Definition of Missing	User-defined missing values are treated as missing.
	Cases Used	Statistics for each table are based on all the cases with valid data in the specified range(s) for all variables in each table.
Syntax		CROSSTABS /TABLES=Lifestyle_Code Supplement_Code Meat_Intake_Code Number_of_Deliveries BY Iron_Deficiency_Code /FORMAT=AVALUE TABLES /STATISTICS=CHISQ RISK /CELLS=COUNT ROW TOTAL.
Resources	Processor Time	00:00:00.02
	Elapsed Time	00:00:00.02
	Dimensions Requested	2
	Cells Available	174762

Case Processing Summary

	Cases					
	Valid		Missing		Total	
	N	Percent	N	Percent	N	Percent
Lifestyle * Iron Deficiency Status	100	100.0%	0	0.0%	100	100.0%
Iron Supplement Use * Iron Deficiency Status	100	100.0%	0	0.0%	100	100.0%
Meat Intake Frequency * Iron Deficiency Status	100	100.0%	0	0.0%	100	100.0%
Number of deliveries * Iron Deficiency Status	100	100.0%	0	0.0%	100	100.0%

Lifestyle * Iron Deficiency Status

Crosstab

			Iron Deficiency Status		Total
			Negative	Positive	
Lifestyle	Active	Count	12	37	49
		% within Lifestyle	24.5%	75.5%	100.0%
		% of Total	12.0%	37.0%	49.0%
	Sedentary	Count	10	41	51
		% within Lifestyle	19.6%	80.4%	100.0%
		% of Total	10.0%	41.0%	51.0%
Total		Count	22	78	100
		% within Lifestyle	22.0%	78.0%	100.0%
		% of Total	22.0%	78.0%	100.0%

Chi-Square Tests

	Value	df	Asymp. Sig. (2-sided)	Exact Sig. (2-sided)	Exact Sig. (1-sided)
Pearson Chi-Square	.347[a]	1	.556		
Continuity Correction[b]	.121	1	.728		
Likelihood Ratio	.347	1	.556		
Fisher's Exact Test				.633	.364
Linear-by-Linear Association	.344	1	.558		
N of Valid Cases	100				

a. 0 cells (0.0%) have expected count less than 5. The minimum expected count is 10.78.

b. Computed only for a 2x2 table

Risk Estimate

	Value	95% Confidence Interval	
		Lower	Upper
Odds Ratio for Lifestyle (Active / Sedentary)	1.330	.514	3.437
For cohort Iron Deficiency Status = Negative	1.249	.595	2.623
For cohort Iron Deficiency Status = Positive	.939	.762	1.158
N of Valid Cases	100		

Iron Supplement Use * Iron Deficiency Status

Crosstab

			Iron Deficiency Status		Total
			Negative	Positive	
Iron Supplement Use	No	Count	12	55	67
		% within Iron Supplement Use	17.9%	82.1%	100.0%
		% of Total	12.0%	55.0%	67.0%
	Yes	Count	10	23	33
		% within Iron Supplement Use	30.3%	69.7%	100.0%
		% of Total	10.0%	23.0%	33.0%
Total		Count	22	78	100
		% within Iron Supplement Use	22.0%	78.0%	100.0%
		% of Total	22.0%	78.0%	100.0%

Chi-Square Tests

	Value	df	Asymp. Sig. (2-sided)	Exact Sig. (2-sided)	Exact Sig. (1-sided)
Pearson Chi-Square	1.979[a]	1	.160		
Continuity Correction[b]	1.322	1	.250		
Likelihood Ratio	1.912	1	.167		
Fisher's Exact Test				.201	.126
Linear-by-Linear Association	1.959	1	.162		
N of Valid Cases	100				

a. 0 cells (0.0%) have expected count less than 5. The minimum expected count is 7.26.

b. Computed only for a 2x2 table

Risk Estimate

	Value	95% Confidence Interval	
		Lower	Upper
Odds Ratio for Iron Supplement Use (No / Yes)	.502	.190	1.324
For cohort Iron Deficiency Status = Negative	.591	.285	1.224
For cohort Iron Deficiency Status = Positive	1.178	.916	1.514
N of Valid Cases	100		

Meat Intake Frequency * Iron Deficiency Status

Crosstab

			Iron Deficiency Status	
			Negative	Positive
Meat Intake Frequency	<3 times/week	Count	15	46
		% within Meat Intake Frequency	24.6%	75.4%
		% of Total	15.0%	46.0%
	?3 times/week	Count	7	32
		% within Meat Intake Frequency	17.9%	82.1%
		% of Total	7.0%	32.0%
Total		Count	22	78
		% within Meat Intake Frequency	22.0%	78.0%
		% of Total	22.0%	78.0%

Crosstab

			Total
Meat Intake Frequency	<3 times/week	Count	61
		% within Meat Intake Frequency	100.0%
		% of Total	61.0%
	?3 times/week	Count	39
		% within Meat Intake Frequency	100.0%
		% of Total	39.0%
Total		Count	100
		% within Meat Intake Frequency	100.0%
		% of Total	100.0%

Chi-Square Tests

	Value	df	Asymp. Sig. (2-sided)	Exact Sig. (2-sided)	Exact Sig. (1-sided)
Pearson Chi-Square	.612[a]	1	.434		
Continuity Correction[b]	.286	1	.593		
Likelihood Ratio	.624	1	.430		
Fisher's Exact Test				.470	.299
Linear-by-Linear Association	.605	1	.437		
N of Valid Cases	100				

a. 0 cells (0.0%) have expected count less than 5. The minimum expected count is 8.58.

b. Computed only for a 2x2 table

Risk Estimate

	Value	95% Confidence Interval	
		Lower	Upper
Odds Ratio for Meat Intake Frequency (<3 times/week / ?3 times/week)	1.491	.546	4.069
For cohort Iron Deficiency Status = Negative	1.370	.614	3.056
For cohort Iron Deficiency Status = Positive	.919	.749	1.128
N of Valid Cases	100		

Number of deliveries * Iron Deficiency Status

Crosstab

			Iron Deficiency Status		Total
			Negative	Positive	
Number of deliveries	.00	Count	13	38	51
		% within Number of deliveries	25.5%	74.5%	100.0%
		% of Total	13.0%	38.0%	51.0%
	1.00	Count	5	13	18
		% within Number of deliveries	27.8%	72.2%	100.0%
		% of Total	5.0%	13.0%	18.0%
	2.00	Count	0	9	9
		% within Number of deliveries	0.0%	100.0%	100.0%
		% of Total	0.0%	9.0%	9.0%
	3.00	Count	1	8	9
		% within Number of deliveries	11.1%	88.9%	100.0%
		% of Total	1.0%	8.0%	9.0%
	4.00	Count	3	10	13
		% within Number of deliveries	23.1%	76.9%	100.0%
		% of Total	3.0%	10.0%	13.0%
Total		Count	22	78	100
		% within Number of deliveries	22.0%	78.0%	100.0%
		% of Total	22.0%	78.0%	100.0%

Chi-Square Tests

	Value	df	Asymp. Sig. (2-sided)
Pearson Chi-Square	3.881[a]	4	.422
Likelihood Ratio	5.886	4	.208
Linear-by-Linear Association	.769	1	.381
N of Valid Cases	100		

a. 4 cells (40.0%) have expected count less than 5. The minimum expected count is 1.98.

Risk Estimate

	Value
Odds Ratio for Number of deliveries (.00 / 1.00)	

a. Risk Estimate statistics cannot be computed. They are only computed for a 2*2 table without empty cells.

```
LOGISTIC REGRESSION VARIABLES Iron_Deficiency_Code
 /METHOD=ENTER BMI_Code Anemia_History_Code Hypertension_Code
   Breastfeeding_Code Lifestyle_Code Supplement_Code Meat_Intake_Code
 /CRITERIA=PIN(0.05) POUT(0.10) ITERATE(20) CUT(0.5).
```

Logistic Regression

Notes

Output Created		04-AUG-2025 03:46:57
Comments		
Input	Filter	<none>
	Weight	<none>
	Split File	<none>
	N of Rows in Working Data File	100
Missing Value Handling	Definition of Missing	User-defined missing values are treated as missing
Syntax		LOGISTIC REGRESSION VARIABLES Iron_Deficiency_Code /METHOD=ENTER BMI_Code Anemia_History_Code Hypertension_Code Breastfeeding_Code Lifestyle_Code Supplement_Code Meat_Intake_Code /CRITERIA=PIN(0.05) POUT(0.10) ITERATE(20) CUT(0.5).
Resources	Processor Time	00:00:00.02
	Elapsed Time	00:00:00.02

Case Processing Summary

Unweighted Cases[a]		N	Percent
Selected Cases	Included in Analysis	100	100.0
	Missing Cases	0	.0
	Total	100	100.0
Unselected Cases		0	.0
Total		100	100.0

a. If weight is in effect, see classification table for the total number of cases.

Dependent Variable Encoding

Original Value	Internal Value
Negative	0
Positive	1

Block 0: Beginning Block

Classification Table[a,b]

Observed			Predicted: Iron Deficiency Status: Negative	Predicted: Iron Deficiency Status: Positive	Percentage Correct
Step 0	Iron Deficiency Status	Negative	0	22	.0
		Positive	0	78	100.0
	Overall Percentage				78.0

a. Constant is included in the model.

b. The cut value is .500

Variables in the Equation

		B	S.E.	Wald	df	Sig.	Exp(B)
Step 0	Constant	1.266	.241	27.489	1	.000	3.545

Variables not in the Equation

			Score	df	Sig.
Step 0	Variables	BMI_Code	13.717	1	.000
		Anemia_History_Code	2.411	1	.120
		Hypertension_Code	4.977	1	.026
		Breastfeeding_Code	.002	1	.961
		Lifestyle_Code	.347	1	.556
		Supplement_Code	1.979	1	.160
		Meat_Intake_Code	.612	1	.434
	Overall Statistics		21.943	7	.003

Block 1: Method = Enter

Omnibus Tests of Model Coefficients

		Chi-square	df	Sig.
Step 1	Step	25.215	7	.001
	Block	25.215	7	.001
	Model	25.215	7	.001

Model Summary

Step	-2 Log likelihood	Cox & Snell R Square	Nagelkerke R Square
1	80.167[a]	.223	.342

a. Estimation terminated at iteration number 20 because maximum iterations has been reached. Final solution cannot be found.

Classification Table[a]

Observed			Predicted: Iron Deficiency Status Negative	Predicted: Iron Deficiency Status Positive	Predicted: Percentage Correct
Step 1	Iron Deficiency Status	Negative	8	14	36.4
		Positive	5	73	93.6
	Overall Percentage				81.0

a. The cut value is .500

Variables in the Equation

		B	S.E.	Wald	df	Sig.
Step 1[a]	BMI_Code	-1.108	.349	10.071	1	.002
	Anemia_History_Code	1.433	.885	2.622	1	.105
	Hypertension_Code	20.332	9652.829	.000	1	.998
	Breastfeeding_Code	-.202	.606	.111	1	.739
	Lifestyle_Code	-.100	.630	.025	1	.874
	Supplement_Code	-.534	.587	.825	1	.364
	Meat_Intake_Code	.324	.638	.259	1	.611
	Constant	2.718	.906	8.998	1	.003

Variables in the Equation

		Exp(B)
Step 1[a]	BMI_Code	.330
	Anemia_History_Code	4.190
	Hypertension_Code	676481340.8
	Breastfeeding_Code	.817
	Lifestyle_Code	.905
	Supplement_Code	.586
	Meat_Intake_Code	1.383
	Constant	15.147

a. Variable(s) entered on step 1: BMI_Code, Anemia_History_Code, Hypertension_Code, Breastfeeding_Code, Lifestyle_Code, Supplement_Code, Meat_Intake_Code.

```
FREQUENCIES VARIABLES=Iron_Deficiency_Code
 /STATISTICS=MODE.
```

Frequencies

Notes

Output Created		04-AUG-2025 03:46:57
Comments		
Input	Filter	<none>
	Weight	<none>
	Split File	<none>
	N of Rows in Working Data File	100
Missing Value Handling	Definition of Missing	User-defined missing values are treated as missing.
	Cases Used	Statistics are based on all cases with valid data.
Syntax		FREQUENCIES VARIABLES=Iron_Deficiency_Code /STATISTICS=MODE.
Resources	Processor Time	00:00:00.02
	Elapsed Time	00:00:00.03

Statistics

Iron Deficiency Status

N	Valid	100
	Missing	0
Mode		1.00

Iron Deficiency Status

		Frequency	Percent	Valid Percent	Cumulative Percent
Valid	Negative	22	22.0	22.0	22.0
	Positive	78	78.0	78.0	100.0
	Total	100	100.0	100.0	

```
MEANS Iron_Deficiency_Code BY BMI_Code
 /STATISTICS=MEAN COUNT.
```

Means

Notes

Output Created		04-AUG-2025 03:46:58
Comments		
Input	Filter	<none>
	Weight	<none>
	Split File	<none>
	N of Rows in Working Data File	100
Missing Value Handling	Definition of Missing	For each dependent variable in a table, user-defined missing values for the dependent and all grouping variables are treated as missing.
	Cases Used	Cases used for each table have no missing values in any independent variable, and not all dependent variables have missing values.
Syntax		MEANS Iron_Deficiency_Code BY BMI_Code /STATISTICS=MEAN COUNT.
Resources	Processor Time	00:00:00.00
	Elapsed Time	00:00:00.00

Warnings

Invalid Statistics subcommand. Found: MEAN
Invalid Statistics subcommand. Found: COUNT

Case Processing Summary

	Cases					
	Included		Excluded		Total	
	N	Percent	N	Percent	N	Percent
Iron Deficiency Status * BMI Category	100	100.0%	0	0.0%	100	100.0%

Report

Iron Deficiency Status

BMI Category	Mean	N	Std. Deviation
Underweight	.8608	79	.34841
Normal	.5000	4	.57735
Overweight/Obese	.4706	17	.51450
Total	.7800	100	.41633

```
MEANS Iron_Deficiency_Code BY Anemia_History_Code
```

```
/STATISTICS=MEAN COUNT.
```

Means

Notes

Output Created		04-AUG-2025 03:46:58
Comments		
Input	Filter	<none>
	Weight	<none>
	Split File	<none>
	N of Rows in Working Data File	100
Missing Value Handling	Definition of Missing	For each dependent variable in a table, user-defined missing values for the dependent and all grouping variables are treated as missing.
	Cases Used	Cases used for each table have no missing values in any independent variable, and not all dependent variables have missing values.
Syntax		MEANS Iron_Deficiency_Code BY Anemia_History_Code /STATISTICS=MEAN COUNT.
Resources	Processor Time	00:00:00.02
	Elapsed Time	00:00:00.02

Warnings

Invalid Statistics subcommand. Found: MEAN
Invalid Statistics subcommand. Found: COUNT

Case Processing Summary

	Cases					
	Included		Excluded		Total	
	N	Percent	N	Percent	N	Percent
Iron Deficiency Status * History of Anemia	100	100.0%	0	0.0%	100	100.0%

Report

Iron Deficiency Status

History of Anemia	Mean	N	Std. Deviation
No	.7468	79	.43760
Yes	.9048	21	.30079
Total	.7800	100	.41633

```
 ECHO "Analysis Complete - Iron Deficiency Study Data Generated Successfully".
Analysis Complete - Iron Deficiency Study Data Generated Successfully
INPUT PROGRAM.
LOOP #I=1 TO 100.
COMPUTE Participant_ID=#I.




COMPUTE Age = TRUNC(UNIFORM(30) + 16).
COMPUTE BMI = NORMAL(22.5) + 3.
IF BMI < 15 BMI = 15.
IF BMI > 35 BMI = 35.
COMPUTE Hemoglobin_g_dL = NORMAL(12.5) + 0.8.
IF Hemoglobin_g_dL < 11.0 Hemoglobin_g_dL = 11.0.
IF Hemoglobin_g_dL > 15.0 Hemoglobin_g_dL = 15.0.
COMPUTE Serum_Ferritin_ug_L = EXP(NORMAL(3.0) + 0.8).
IF Serum_Ferritin_ug_L > 150 Serum_Ferritin_ug_L = 150.
COMPUTE Serum_Iron_umol_L = NORMAL(15) + 5.
IF Serum_Iron_umol_L < 5 Serum_Iron_umol_L = 5.
IF Serum_Iron_umol_L > 30 Serum_Iron_umol_L = 30.


COMPUTE Age_Group_Code = 2.
IF Age <= 25 Age_Group_Code = 1.
IF Age >= 36 Age_Group_Code = 3.

COMPUTE Education_Code = 2.
IF UNIFORM(1) < 0.26 Education_Code = 1.
IF UNIFORM(1) < 0.32 Education_Code = 3.

COMPUTE BMI_Code = 2.
IF BMI < 18.5 BMI_Code = 1.
IF BMI >= 25 BMI_Code = 3.

COMPUTE Residence_Code = 2.
IF UNIFORM(1) < 0.38 Residence_Code = 1.

COMPUTE Marital_Code = 1.
IF UNIFORM(1) < 0.31 Marital_Code = 0.

COMPUTE Anemia_History_Code = 0.
IF UNIFORM(1) < 0.27 Anemia_History_Code = 1.

COMPUTE Hypertension_Code = 0.
IF UNIFORM(1) < 0.21 Hypertension_Code = 1.

COMPUTE Diabetes_Code = 0.
IF UNIFORM(1) < 0.13 Diabetes_Code = 1.

COMPUTE Breastfeeding_Code = 0.
```

```
COMPUTE Smoking_Code = 0.
IF UNIFORM(1) < 0.09 Smoking_Code = 1.

COMPUTE Contraceptive_Code = 0.
IF UNIFORM(1) < 0.36 Contraceptive_Code = 1.

COMPUTE Lifestyle_Code = 1.
IF UNIFORM(1) < 0.45 Lifestyle_Code = 0.

COMPUTE Number_of_Deliveries = 0.
IF Marital_Code = 1 AND UNIFORM(1) < 0.7 Number_of_Deliveries = TRUNC(UNIFORM(4) + 1).

COMPUTE Supplement_Code = 1.
IF UNIFORM(1) < 0.64 Supplement_Code = 0.

COMPUTE Meat_Intake_Code = 1.
IF UNIFORM(1) < 0.61 Meat_Intake_Code = 0.

COMPUTE Doctor_Visits_Code = 1.
IF UNIFORM(1) < 0.52 Doctor_Visits_Code = 0.


COMPUTE Iron_Deficiency_Code = 0.
IF (Serum_Ferritin_ug_L < 15 AND Serum_Iron_umol_L < 10) Iron_Deficiency_Code = 1.


IF (BMI_Code = 1 AND UNIFORM(1) < 0.8) Iron_Deficiency_Code = 1.
IF (Anemia_History_Code = 1 AND UNIFORM(1) < 0.77) Iron_Deficiency_Code = 1.
IF (Hypertension_Code = 1 AND UNIFORM(1) < 0.66) Iron_Deficiency_Code = 1.

END CASE.
END LOOP.
END FILE.
END INPUT PROGRAM.

EXECUTE.


VARIABLE LABELS
  Participant_ID 'Participant ID'
  Age 'Age in years'
  BMI 'Body Mass Index (kg/m²)'
  Hemoglobin_g_dL 'Hemoglobin level (g/dL)'
  Serum_Ferritin_ug_L 'Serum Ferritin (µg/L)'
  Serum_Iron_umol_L 'Serum Iron (µmol/L)'
  Age_Group_Code 'Age Group'
  Education_Code 'Education Level'
  BMI_Code 'BMI Category'
  Residence_Code 'Residence'
  Marital_Code 'Marital Status'
  Anemia_History_Code 'History of Anemia'
  Hypertension_Code 'Hypertension'
```

```
  Diabetes_Code 'Diabetes'
  Breastfeeding_Code 'Breastfeeding History'
  Smoking_Code 'Smoking History'
  Contraceptive_Code 'Contraceptive Use'
  Lifestyle_Code 'Lifestyle'
  Number_of_Deliveries 'Number of deliveries'
  Supplement_Code 'Iron Supplement Use'
  Meat_Intake_Code 'Meat Intake Frequency'
  Doctor_Visits_Code 'Doctor Visit Frequency'
  Iron_Deficiency_Code 'Iron Deficiency Status'.

VALUE LABELS
  Age_Group_Code 1 '16-25 years' 2 '26-35 years' 3 '36-45 years'
  /Education_Code 1 'Below Secondary' 2 'Secondary' 3 'Graduate+'
  /BMI_Code 1 'Underweight' 2 'Normal' 3 'Overweight/Obese'
  /Residence_Code 1 'Rural' 2 'Urban'
  /Marital_Code 0 'Unmarried' 1 'Married'
  /Anemia_History_Code 0 'No' 1 'Yes'
  /Hypertension_Code 0 'No' 1 'Yes'
  /Diabetes_Code 0 'No' 1 'Yes'
  /Breastfeeding_Code 0 'No' 1 'Yes'
  /Smoking_Code 0 'No' 1 'Yes'
  /Contraceptive_Code 0 'No' 1 'Yes'
  /Lifestyle_Code 0 'Active' 1 'Sedentary'
  /Supplement_Code 0 'No' 1 'Yes'
  /Meat_Intake_Code 0 '<3 times/week' 1 '?3 times/week'
  /Doctor_Visits_Code 0 '<1/month' 1 '?1/month'
  /Iron_Deficiency_Code 0 'Negative' 1 'Positive'.

MISSING VALUES
  Age (99) BMI (99.9) Hemoglobin_g_dL (99.9) Serum_Ferritin_ug_L (999.9)
  Serum_Iron_umol_L (999.9) Number_of_Deliveries (9)
  Age_Group_Code Education_Code BMI_Code Residence_Code Marital_Code
  Anemia_History_Code Hypertension_Code Diabetes_Code Breastfeeding_Code
  Smoking_Code Contraceptive_Code Lifestyle_Code Supplement_Code
  Meat_Intake_Code Doctor_Visits_Code Iron_Deficiency_Code (9).
```

```
FREQUENCIES VARIABLES=BMI_Code Anemia_History_Code Hypertension_Code Iron_Deficiency_Code
  /ORDER=ANALYSIS.
```

Frequencies

Notes

Output Created		04-AUG-2025 13:19:45
Comments		
Input	Filter	<none>
	Weight	<none>
	Split File	<none>
	N of Rows in Working Data File	100
Missing Value Handling	Definition of Missing	User-defined missing values are treated as missing.
	Cases Used	Statistics are based on all cases with valid data.
Syntax		FREQUENCIES VARIABLES=BMI_Code Anemia_History_Code Hypertension_Code Iron_Deficiency_Code /ORDER=ANALYSIS.
Resources	Processor Time	00:00:00.00
	Elapsed Time	00:00:00.00

Statistics

		BMI Category	History of Anemia	Hypertension	Iron Deficiency Status
N	Valid	100	100	100	100
	Missing	0	0	0	0

Frequency Table

BMI Category

		Frequency	Percent	Valid Percent	Cumulative Percent
Valid	Underweight	74	74.0	74.0	74.0
	Normal	11	11.0	11.0	85.0
	Overweight/Obese	15	15.0	15.0	100.0
	Total	100	100.0	100.0	

History of Anemia

		Frequency	Percent	Valid Percent	Cumulative Percent
Valid	No	71	71.0	71.0	71.0
	Yes	29	29.0	29.0	100.0
	Total	100	100.0	100.0	

Hypertension

		Frequency	Percent	Valid Percent	Cumulative Percent
Valid	No	77	77.0	77.0	77.0
	Yes	23	23.0	23.0	100.0
	Total	100	100.0	100.0	

Iron Deficiency Status

		Frequency	Percent	Valid Percent	Cumulative Percent
Valid	Negative	12	12.0	12.0	12.0
	Positive	88	88.0	88.0	100.0
	Total	100	100.0	100.0	

```
DESCRIPTIVES VARIABLES=BMI_Code Anemia_History_Code Hypertension_Code Iron_Deficiency_Code
  /STATISTICS=MEAN STDDEV MIN MAX.
```

Descriptives

Notes

Output Created		04-AUG-2025 13:19:46
Comments		
Input	Filter	<none>
	Weight	<none>
	Split File	<none>
	N of Rows in Working Data File	100
Missing Value Handling	Definition of Missing	User defined missing values are treated as missing.
	Cases Used	All non-missing data are used.
Syntax		DESCRIPTIVES VARIABLES=BMI_Code Anemia_History_Code Hypertension_Code Iron_Deficiency_Code /STATISTICS=MEAN STDDEV MIN MAX.
Resources	Processor Time	00:00:00.00
	Elapsed Time	00:00:00.00

Descriptive Statistics

	N	Minimum	Maximum	Mean	Std. Deviation
BMI Category	100	1.00	3.00	1.4100	.73985
History of Anemia	100	.00	1.00	.2900	.45605
Hypertension	100	.00	1.00	.2300	.42295
Iron Deficiency Status	100	.00	1.00	.8800	.32660
Valid N (listwise)	100				

```
CROSSTABS
  /TABLES=BMI_Code Anemia_History_Code Hypertension_Code BY Iron_Deficiency_Code
  /FORMAT=AVALUE TABLES
  /STATISTICS=CHISQ PHI CC
  /CELLS=COUNT ROW COLUMN TOTAL.
```

Crosstabs

Notes

Output Created		04-AUG-2025 13:19:46
Comments		
Input	Filter	<none>
	Weight	<none>
	Split File	<none>
	N of Rows in Working Data File	100
Missing Value Handling	Definition of Missing	User-defined missing values are treated as missing.
	Cases Used	Statistics for each table are based on all the cases with valid data in the specified range(s) for all variables in each table.
Syntax		CROSSTABS /TABLES=BMI_Code Anemia_History_Code Hypertension_Code BY Iron_Deficiency_Code /FORMAT=AVALUE TABLES /STATISTICS=CHISQ PHI CC /CELLS=COUNT ROW COLUMN TOTAL.
Resources	Processor Time	00:00:00.02
	Elapsed Time	00:00:00.02
	Dimensions Requested	2
	Cells Available	174762

Case Processing Summary

	Cases					
	Valid		Missing		Total	
	N	Percent	N	Percent	N	Percent
BMI Category * Iron Deficiency Status	100	100.0%	0	0.0%	100	100.0%
History of Anemia * Iron Deficiency Status	100	100.0%	0	0.0%	100	100.0%
Hypertension * Iron Deficiency Status	100	100.0%	0	0.0%	100	100.0%

BMI Category * Iron Deficiency Status

Crosstab

			Iron Deficiency Status		Total
			Negative	Positive	
BMI Category	Underweight	Count	4	70	74
		% within BMI Category	5.4%	94.6%	100.0%
		% within Iron Deficiency Status	33.3%	79.5%	74.0%
		% of Total	4.0%	70.0%	74.0%
	Normal	Count	3	8	11
		% within BMI Category	27.3%	72.7%	100.0%
		% within Iron Deficiency Status	25.0%	9.1%	11.0%
		% of Total	3.0%	8.0%	11.0%
	Overweight/Obese	Count	5	10	15
		% within BMI Category	33.3%	66.7%	100.0%
		% within Iron Deficiency Status	41.7%	11.4%	15.0%
		% of Total	5.0%	10.0%	15.0%
Total		Count	12	88	100
		% within BMI Category	12.0%	88.0%	100.0%
		% within Iron Deficiency Status	100.0%	100.0%	100.0%
		% of Total	12.0%	88.0%	100.0%

Chi-Square Tests

	Value	df	Asymp. Sig. (2-sided)
Pearson Chi-Square	11.942[a]	2	.003
Likelihood Ratio	10.277	2	.006
Linear-by-Linear Association	11.295	1	.001
N of Valid Cases	100		

a. 2 cells (33.3%) have expected count less than 5. The minimum expected count is 1.32.

Symmetric Measures

		Value	Approx. Sig.
Nominal by Nominal	Phi	.346	.003
	Cramer's V	.346	.003
	Contingency Coefficient	.327	.003
N of Valid Cases		100	

a. Not assuming the null hypothesis.

b. Using the asymptotic standard error assuming the null hypothesis.

History of Anemia * Iron Deficiency Status

Crosstab

			Iron Deficiency Status		
			Negative	Positive	Total
History of Anemia	No	Count	12	59	71
		% within History of Anemia	16.9%	83.1%	100.0%
		% within Iron Deficiency Status	100.0%	67.0%	71.0%
		% of Total	12.0%	59.0%	71.0%
	Yes	Count	0	29	29
		% within History of Anemia	0.0%	100.0%	100.0%
		% within Iron Deficiency Status	0.0%	33.0%	29.0%
		% of Total	0.0%	29.0%	29.0%
Total		Count	12	88	100
		% within History of Anemia	12.0%	88.0%	100.0%
		% within Iron Deficiency Status	100.0%	100.0%	100.0%
		% of Total	12.0%	88.0%	100.0%

Chi-Square Tests

	Value	df	Asymp. Sig. (2-sided)	Exact Sig. (2-sided)	Exact Sig. (1-sided)
Pearson Chi-Square	5.570[a]	1	.018		
Continuity Correction[b]	4.084	1	.043		
Likelihood Ratio	8.872	1	.003		
Fisher's Exact Test				.017	.012
Linear-by-Linear Association	5.514	1	.019		
N of Valid Cases	100				

a. 1 cells (25.0%) have expected count less than 5. The minimum expected count is 3.48.

b. Computed only for a 2x2 table

Symmetric Measures

		Value	Approx. Sig.
Nominal by Nominal	Phi	.236	.018
	Cramer's V	.236	.018
	Contingency Coefficient	.230	.018
N of Valid Cases		100	

a. Not assuming the null hypothesis.

b. Using the asymptotic standard error assuming the null hypothesis.

Hypertension * Iron Deficiency Status

Crosstab

			Iron Deficiency Status		
			Negative	Positive	Total
Hypertension	No	Count	12	65	77
		% within Hypertension	15.6%	84.4%	100.0%
		% within Iron Deficiency Status	100.0%	73.9%	77.0%
		% of Total	12.0%	65.0%	77.0%
	Yes	Count	0	23	23
		% within Hypertension	0.0%	100.0%	100.0%
		% within Iron Deficiency Status	0.0%	26.1%	23.0%
		% of Total	0.0%	23.0%	23.0%
Total		Count	12	88	100
		% within Hypertension	12.0%	88.0%	100.0%
		% within Iron Deficiency Status	100.0%	100.0%	100.0%
		% of Total	12.0%	88.0%	100.0%

Chi-Square Tests

	Value	df	Asymp. Sig. (2-sided)	Exact Sig. (2-sided)	Exact Sig. (1-sided)
Pearson Chi-Square	4.073[a]	1	.044		
Continuity Correction[b]	2.731	1	.098		
Likelihood Ratio	6.747	1	.009		
Fisher's Exact Test				.063	.035
Linear-by-Linear Association	4.032	1	.045		
N of Valid Cases	100				

a. 1 cells (25.0%) have expected count less than 5. The minimum expected count is 2.76.

b. Computed only for a 2x2 table

Symmetric Measures

		Value	Approx. Sig.
Nominal by Nominal	Phi	.202	.044
	Cramer's V	.202	.044
	Contingency Coefficient	.198	.044
N of Valid Cases		100	

a. Not assuming the null hypothesis.

b. Using the asymptotic standard error assuming the null hypothesis.

```
CROSSTABS
  /TABLES=BMI_Code BY Iron_Deficiency_Code
  /FORMAT=AVALUE TABLES
  /STATISTICS=CHISQ RISK
  /CELLS=COUNT ROW TOTAL.
```

Crosstabs

Notes

Output Created		04-AUG-2025 13:19:46
Comments		
Input	Filter	<none>
	Weight	<none>
	Split File	<none>
	N of Rows in Working Data File	100
Missing Value Handling	Definition of Missing	User-defined missing values are treated as missing.
	Cases Used	Statistics for each table are based on all the cases with valid data in the specified range(s) for all variables in each table.
Syntax		CROSSTABS /TABLES=BMI_Code BY Iron_Deficiency_Code /FORMAT=AVALUE TABLES /STATISTICS=CHISQ RISK /CELLS=COUNT ROW TOTAL.
Resources	Processor Time	00:00:00.02
	Elapsed Time	00:00:00.02
	Dimensions Requested	2
	Cells Available	174762

Case Processing Summary

	Cases					
	Valid		Missing		Total	
	N	Percent	N	Percent	N	Percent
BMI Category * Iron Deficiency Status	100	100.0%	0	0.0%	100	100.0%

BMI Category * Iron Deficiency Status Crosstabulation

			Iron Deficiency Status		Total
			Negative	Positive	
BMI Category	Underweight	Count	4	70	74
		% within BMI Category	5.4%	94.6%	100.0%
		% of Total	4.0%	70.0%	74.0%
	Normal	Count	3	8	11
		% within BMI Category	27.3%	72.7%	100.0%
		% of Total	3.0%	8.0%	11.0%
	Overweight/Obese	Count	5	10	15
		% within BMI Category	33.3%	66.7%	100.0%
		% of Total	5.0%	10.0%	15.0%
Total		Count	12	88	100
		% within BMI Category	12.0%	88.0%	100.0%
		% of Total	12.0%	88.0%	100.0%

Chi-Square Tests

	Value	df	Asymp. Sig. (2-sided)
Pearson Chi-Square	11.942[a]	2	.003
Likelihood Ratio	10.277	2	.006
Linear-by-Linear Association	11.295	1	.001
N of Valid Cases	100		

a. 2 cells (33.3%) have expected count less than 5. The minimum expected count is 1.32.

Risk Estimate

	Value
Odds Ratio for BMI Category (Underweight / Normal)	

a. Risk Estimate statistics cannot be computed. They are only computed for a 2*2 table without empty cells.

CROSSTABS

```
/TABLES=Anemia_History_Code BY Iron_Deficiency_Code
/FORMAT=AVALUE TABLES
/STATISTICS=CHISQ RISK
/CELLS=COUNT ROW TOTAL.
```

Crosstabs

Notes

Output Created		04-AUG-2025 13:19:46
Comments		
Input	Filter	<none>
	Weight	<none>
	Split File	<none>
	N of Rows in Working Data File	100
Missing Value Handling	Definition of Missing	User-defined missing values are treated as missing.
	Cases Used	Statistics for each table are based on all the cases with valid data in the specified range(s) for all variables in each table.
Syntax		CROSSTABS<br><br>/TABLES=Anemia_History_Code BY Iron_Deficiency_Code<br>/FORMAT=AVALUE TABLES<br>/STATISTICS=CHISQ RISK<br>/CELLS=COUNT ROW TOTAL.
Resources	Processor Time	00:00:00.00
	Elapsed Time	00:00:00.01
	Dimensions Requested	2
	Cells Available	174762

Case Processing Summary

	Cases					
	Valid		Missing		Total	
	N	Percent	N	Percent	N	Percent
History of Anemia * Iron Deficiency Status	100	100.0%	0	0.0%	100	100.0%

History of Anemia * Iron Deficiency Status Crosstabulation

			Iron Deficiency Status		Total
			Negative	Positive	
History of Anemia	No	Count	12	59	71
		% within History of Anemia	16.9%	83.1%	100.0%
		% of Total	12.0%	59.0%	71.0%
	Yes	Count	0	29	29
		% within History of Anemia	0.0%	100.0%	100.0%
		% of Total	0.0%	29.0%	29.0%
Total		Count	12	88	100
		% within History of Anemia	12.0%	88.0%	100.0%
		% of Total	12.0%	88.0%	100.0%

Chi-Square Tests

	Value	df	Asymp. Sig. (2-sided)	Exact Sig. (2-sided)	Exact Sig. (1-sided)
Pearson Chi-Square	5.570[a]	1	.018		
Continuity Correction[b]	4.084	1	.043		
Likelihood Ratio	8.872	1	.003		
Fisher's Exact Test				.017	.012
Linear-by-Linear Association	5.514	1	.019		
N of Valid Cases	100				

a. 1 cells (25.0%) have expected count less than 5. The minimum expected count is 3.48.

b. Computed only for a 2x2 table

Risk Estimate

	Value	95% Confidence Interval	
		Lower	Upper
For cohort Iron Deficiency Status = Positive	.831	.748	.923
N of Valid Cases	100		

```
CROSSTABS
 /TABLES=Hypertension_Code BY Iron_Deficiency_Code
 /FORMAT=AVALUE TABLES
 /STATISTICS=CHISQ RISK
 /CELLS=COUNT ROW TOTAL.
```

Crosstabs

Notes

Output Created		04-AUG-2025 13:19:46
Comments		
Input	Filter	<none>
	Weight	<none>
	Split File	<none>
	N of Rows in Working Data File	100
Missing Value Handling	Definition of Missing	User-defined missing values are treated as missing.
	Cases Used	Statistics for each table are based on all the cases with valid data in the specified range(s) for all variables in each table.
Syntax		CROSSTABS /TABLES=Hypertension_Code BY Iron_Deficiency_Code /FORMAT=AVALUE TABLES /STATISTICS=CHISQ RISK /CELLS=COUNT ROW TOTAL.
Resources	Processor Time	00:00:00.02
	Elapsed Time	00:00:00.02
	Dimensions Requested	2
	Cells Available	174762

Case Processing Summary

	Cases					
	Valid		Missing		Total	
	N	Percent	N	Percent	N	Percent
Hypertension * Iron Deficiency Status	100	100.0%	0	0.0%	100	100.0%

Hypertension * Iron Deficiency Status Crosstabulation

			Iron Deficiency Status		Total
			Negative	Positive	
Hypertension	No	Count	12	65	77
		% within Hypertension	15.6%	84.4%	100.0%
		% of Total	12.0%	65.0%	77.0%
	Yes	Count	0	23	23
		% within Hypertension	0.0%	100.0%	100.0%
		% of Total	0.0%	23.0%	23.0%
Total		Count	12	88	100
		% within Hypertension	12.0%	88.0%	100.0%
		% of Total	12.0%	88.0%	100.0%

Chi-Square Tests

	Value	df	Asymp. Sig. (2-sided)	Exact Sig. (2-sided)	Exact Sig. (1-sided)
Pearson Chi-Square	4.073[a]	1	.044		
Continuity Correction[b]	2.731	1	.098		
Likelihood Ratio	6.747	1	.009		
Fisher's Exact Test				.063	.035
Linear-by-Linear Association	4.032	1	.045		
N of Valid Cases	100				

a. 1 cells (25.0%) have expected count less than 5. The minimum expected count is 2.76.

b. Computed only for a 2x2 table

Risk Estimate

	Value	95% Confidence Interval	
		Lower	Upper
For cohort Iron Deficiency Status = Positive	.844	.767	.929
N of Valid Cases	100		

```
CROSSTABS
 /TABLES=Lifestyle_Code Supplement_Code Meat_Intake_Code Number_of_Deliveries BY Iron_Defic
 /FORMAT=AVALUE TABLES
 /STATISTICS=CHISQ RISK
 /CELLS=COUNT ROW TOTAL.
```

Crosstabs

Notes

Output Created		04-AUG-2025 13:19:46
Comments		
Input	Filter	<none>
	Weight	<none>
	Split File	<none>
	N of Rows in Working Data File	100
Missing Value Handling	Definition of Missing	User-defined missing values are treated as missing.
	Cases Used	Statistics for each table are based on all the cases with valid data in the specified range(s) for all variables in each table.
Syntax		CROSSTABS /TABLES=Lifestyle_Code Supplement_Code Meat_Intake_Code Number_of_Deliveries BY Iron_Deficiency_Code /FORMAT=AVALUE TABLES /STATISTICS=CHISQ RISK /CELLS=COUNT ROW TOTAL.
Resources	Processor Time	00:00:00.02
	Elapsed Time	00:00:00.01
	Dimensions Requested	2
	Cells Available	174762

Case Processing Summary

	Cases					
	Valid		Missing		Total	
	N	Percent	N	Percent	N	Percent
Lifestyle * Iron Deficiency Status	100	100.0%	0	0.0%	100	100.0%
Iron Supplement Use * Iron Deficiency Status	100	100.0%	0	0.0%	100	100.0%
Meat Intake Frequency * Iron Deficiency Status	100	100.0%	0	0.0%	100	100.0%
Number of deliveries * Iron Deficiency Status	100	100.0%	0	0.0%	100	100.0%

Lifestyle * Iron Deficiency Status

Crosstab

			Iron Deficiency Status		Total
			Negative	Positive	
Lifestyle	Active	Count	7	43	50
		% within Lifestyle	14.0%	86.0%	100.0%
		% of Total	7.0%	43.0%	50.0%
	Sedentary	Count	5	45	50
		% within Lifestyle	10.0%	90.0%	100.0%
		% of Total	5.0%	45.0%	50.0%
Total		Count	12	88	100
		% within Lifestyle	12.0%	88.0%	100.0%
		% of Total	12.0%	88.0%	100.0%

Chi-Square Tests

	Value	df	Asymp. Sig. (2-sided)	Exact Sig. (2-sided)	Exact Sig. (1-sided)
Pearson Chi-Square	.379[a]	1	.538		
Continuity Correction[b]	.095	1	.758		
Likelihood Ratio	.380	1	.537		
Fisher's Exact Test				.760	.380
Linear-by-Linear Association	.375	1	.540		
N of Valid Cases	100				

a. 0 cells (0.0%) have expected count less than 5. The minimum expected count is 6.00.

b. Computed only for a 2x2 table

Risk Estimate

	Value	95% Confidence Interval	
		Lower	Upper
Odds Ratio for Lifestyle (Active / Sedentary)	1.465	.432	4.969
For cohort Iron Deficiency Status = Negative	1.400	.476	4.117
For cohort Iron Deficiency Status = Positive	.956	.827	1.105
N of Valid Cases	100		

Iron Supplement Use * Iron Deficiency Status

Crosstab

			Iron Deficiency Status		Total
			Negative	Positive	
Iron Supplement Use	No	Count	7	61	68
		% within Iron Supplement Use	10.3%	89.7%	100.0%
		% of Total	7.0%	61.0%	68.0%
	Yes	Count	5	27	32
		% within Iron Supplement Use	15.6%	84.4%	100.0%
		% of Total	5.0%	27.0%	32.0%
Total		Count	12	88	100
		% within Iron Supplement Use	12.0%	88.0%	100.0%
		% of Total	12.0%	88.0%	100.0%

Chi-Square Tests

	Value	df	Asymp. Sig. (2-sided)	Exact Sig. (2-sided)	Exact Sig. (1-sided)
Pearson Chi-Square	.586[a]	1	.444		
Continuity Correction[b]	.190	1	.663		
Likelihood Ratio	.564	1	.453		
Fisher's Exact Test				.514	.323
Linear-by-Linear Association	.580	1	.446		
N of Valid Cases	100				

a. 1 cells (25.0%) have expected count less than 5. The minimum expected count is 3.84.

b. Computed only for a 2x2 table

Risk Estimate

	Value	95% Confidence Interval	
		Lower	Upper
Odds Ratio for Iron Supplement Use (No / Yes)	.620	.180	2.128
For cohort Iron Deficiency Status = Negative	.659	.226	1.917
For cohort Iron Deficiency Status = Positive	1.063	.897	1.260
N of Valid Cases	100		

Meat Intake Frequency * Iron Deficiency Status

Crosstab

			Iron Deficiency Status	
			Negative	Positive
Meat Intake Frequency	<3 times/week	Count	8	46
		% within Meat Intake Frequency	14.8%	85.2%
		% of Total	8.0%	46.0%
	?3 times/week	Count	4	42
		% within Meat Intake Frequency	8.7%	91.3%
		% of Total	4.0%	42.0%
Total		Count	12	88
		% within Meat Intake Frequency	12.0%	88.0%
		% of Total	12.0%	88.0%

Crosstab

			Total
Meat Intake Frequency	<3 times/week	Count	54
		% within Meat Intake Frequency	100.0%
		% of Total	54.0%
	?3 times/week	Count	46
		% within Meat Intake Frequency	100.0%
		% of Total	46.0%
Total		Count	100
		% within Meat Intake Frequency	100.0%
		% of Total	100.0%

Chi-Square Tests

	Value	df	Asymp. Sig. (2-sided)	Exact Sig. (2-sided)	Exact Sig. (1-sided)
Pearson Chi-Square	.881[a]	1	.348		
Continuity Correction[b]	.397	1	.529		
Likelihood Ratio	.900	1	.343		
Fisher's Exact Test				.539	.267
Linear-by-Linear Association	.872	1	.350		
N of Valid Cases	100				

a. 0 cells (0.0%) have expected count less than 5. The minimum expected count is 5.52.

b. Computed only for a 2x2 table

Risk Estimate

	Value	95% Confidence Interval	
		Lower	Upper
Odds Ratio for Meat Intake Frequency (<3 times/week / ?3 times/week)	1.826	.512	6.509
For cohort Iron Deficiency Status = Negative	1.704	.548	5.295
For cohort Iron Deficiency Status = Positive	.933	.809	1.076
N of Valid Cases	100		

Number of deliveries * Iron Deficiency Status

Crosstab

			Iron Deficiency Status		Total
			Negative	Positive	
Number of deliveries	.00	Count	6	50	56
		% within Number of deliveries	10.7%	89.3%	100.0%
		% of Total	6.0%	50.0%	56.0%
	1.00	Count	2	8	10
		% within Number of deliveries	20.0%	80.0%	100.0%
		% of Total	2.0%	8.0%	10.0%
	2.00	Count	3	13	16
		% within Number of deliveries	18.8%	81.2%	100.0%
		% of Total	3.0%	13.0%	16.0%
	3.00	Count	0	8	8
		% within Number of deliveries	0.0%	100.0%	100.0%
		% of Total	0.0%	8.0%	8.0%
	4.00	Count	1	9	10
		% within Number of deliveries	10.0%	90.0%	100.0%
		% of Total	1.0%	9.0%	10.0%
Total		Count	12	88	100
		% within Number of deliveries	12.0%	88.0%	100.0%
		% of Total	12.0%	88.0%	100.0%

Chi-Square Tests

	Value	df	Asymp. Sig. (2-sided)
Pearson Chi-Square	2.513[a]	4	.642
Likelihood Ratio	3.297	4	.509
Linear-by-Linear Association	.025	1	.874
N of Valid Cases	100		

a. 4 cells (40.0%) have expected count less than 5. The minimum expected count is .96.

Risk Estimate

	Value
Odds Ratio for Number of deliveries (.00 / 1.00)	

a. Risk Estimate statistics cannot be computed. They are only computed for a 2*2 table without empty cells.

```
LOGISTIC REGRESSION VARIABLES Iron_Deficiency_Code
 /METHOD=ENTER BMI_Code Anemia_History_Code Hypertension_Code
   Breastfeeding_Code Lifestyle_Code Supplement_Code Meat_Intake_Code
 /CRITERIA=PIN(0.05) POUT(0.10) ITERATE(20) CUT(0.5).
```

Logistic Regression

Notes

Output Created		04-AUG-2025 13:19:46
Comments		
Input	Filter	<none>
	Weight	<none>
	Split File	<none>
	N of Rows in Working Data File	100
Missing Value Handling	Definition of Missing	User-defined missing values are treated as missing
Syntax		LOGISTIC REGRESSION VARIABLES Iron_Deficiency_Code /METHOD=ENTER BMI_Code Anemia_History_Code Hypertension_Code Breastfeeding_Code Lifestyle_Code Supplement_Code Meat_Intake_Code /CRITERIA=PIN(0.05) POUT(0.10) ITERATE(20) CUT(0.5).
Resources	Processor Time	00:00:00.00
	Elapsed Time	00:00:00.00

Case Processing Summary

Unweighted Cases[a]		N	Percent
Selected Cases	Included in Analysis	100	100.0
	Missing Cases	0	.0
	Total	100	100.0
Unselected Cases		0	.0
Total		100	100.0

a. If weight is in effect, see classification table for the total number of cases.

Dependent Variable Encoding

Original Value	Internal Value
Negative	0
Positive	1

Block 0: Beginning Block

Classification Table[a,b]

Observed			Predicted: Iron Deficiency Status: Negative	Predicted: Iron Deficiency Status: Positive	Percentage Correct
Step 0	Iron Deficiency Status	Negative	0	12	.0
		Positive	0	88	100.0
	Overall Percentage				88.0

a. Constant is included in the model.

b. The cut value is .500

Variables in the Equation

		B	S.E.	Wald	df	Sig.	Exp(B)
Step 0	Constant	1.992	.308	41.921	1	.000	7.333

Variables not in the Equation

			Score	df	Sig.
Step 0	Variables	BMI_Code	11.409	1	.001
		Anemia_History_Code	5.570	1	.018
		Hypertension_Code	4.073	1	.044
		Breastfeeding_Code	.273	1	.602
		Lifestyle_Code	.379	1	.538
		Supplement_Code	.586	1	.444
		Meat_Intake_Code	.881	1	.348
	Overall Statistics		22.226	7	.002

Block 1: Method = Enter

Omnibus Tests of Model Coefficients

		Chi-square	df	Sig.
Step 1	Step	27.692	7	.000
	Block	27.692	7	.000
	Model	27.692	7	.000

Model Summary

Step	-2 Log likelihood	Cox & Snell R Square	Nagelkerke R Square
1	45.693[a]	.242	.465

a. Estimation terminated at iteration number 20 because maximum iterations has been reached. Final solution cannot be found.

Classification Table[a]

Observed			Predicted: Iron Deficiency Status: Negative	Predicted: Iron Deficiency Status: Positive	Predicted: Percentage Correct
Step 1	Iron Deficiency Status	Negative	4	8	33.3
		Positive	4	84	95.5
	Overall Percentage				88.0

a. The cut value is .500

Variables in the Equation

		B	S.E.	Wald	df	Sig.
Step 1[a]	BMI_Code	-1.389	.478	8.453	1	.004
	Anemia_History_Code	19.464	6699.352	.000	1	.998
	Hypertension_Code	19.697	7262.002	.000	1	.998
	Breastfeeding_Code	-.222	.797	.078	1	.780
	Lifestyle_Code	.479	.780	.377	1	.539
	Supplement_Code	-.886	.786	1.272	1	.259
	Meat_Intake_Code	.575	.798	.520	1	.471
	Constant	3.554	1.265	7.893	1	.005

Variables in the Equation

		Exp(B)
Step 1[a]	BMI_Code	.249
	Anemia_History_Code	283945111.0
	Hypertension_Code	358215639.5
	Breastfeeding_Code	.801
	Lifestyle_Code	1.615
	Supplement_Code	.412
	Meat_Intake_Code	1.777
	Constant	34.941

a. Variable(s) entered on step 1: BMI_Code, Anemia_History_Code, Hypertension_Code, Breastfeeding_Code, Lifestyle_Code, Supplement_Code, Meat_Intake_Code.

```
FREQUENCIES VARIABLES=Iron_Deficiency_Code
 /STATISTICS=MODE.
```

Frequencies

Notes

Output Created		04-AUG-2025 13:19:46
Comments		
Input	Filter	<none>
	Weight	<none>
	Split File	<none>
	N of Rows in Working Data File	100
Missing Value Handling	Definition of Missing	User-defined missing values are treated as missing.
	Cases Used	Statistics are based on all cases with valid data.
Syntax		FREQUENCIES VARIABLES=Iron_Deficiency_Code /STATISTICS=MODE.
Resources	Processor Time	00:00:00.02
	Elapsed Time	00:00:00.02

Statistics

Iron Deficiency Status

N	Valid	100
	Missing	0
Mode		1.00

Iron Deficiency Status

		Frequency	Percent	Valid Percent	Cumulative Percent
Valid	Negative	12	12.0	12.0	12.0
	Positive	88	88.0	88.0	100.0
	Total	100	100.0	100.0	

```
MEANS Iron_Deficiency_Code BY BMI_Code
 /STATISTICS=MEAN COUNT.
```

Means

Notes

Output Created		04-AUG-2025 13:19:46
Comments		
Input	Filter	<none>
	Weight	<none>
	Split File	<none>
	N of Rows in Working Data File	100
Missing Value Handling	Definition of Missing	For each dependent variable in a table, user-defined missing values for the dependent and all grouping variables are treated as missing.
	Cases Used	Cases used for each table have no missing values in any independent variable, and not all dependent variables have missing values.
Syntax		MEANS Iron_Deficiency_Code BY BMI_Code /STATISTICS=MEAN COUNT.
Resources	Processor Time	00:00:00.00
	Elapsed Time	00:00:00.00

Warnings

Invalid Statistics subcommand. Found: MEAN
Invalid Statistics subcommand. Found: COUNT

Case Processing Summary

	Cases					
	Included		Excluded		Total	
	N	Percent	N	Percent	N	Percent
Iron Deficiency Status * BMI Category	100	100.0%	0	0.0%	100	100.0%

Report

Iron Deficiency Status

BMI Category	Mean	N	Std. Deviation
Underweight	.9459	74	.22767
Normal	.7273	11	.46710
Overweight/Obese	.6667	15	.48795
Total	.8800	100	.32660

```
MEANS Iron_Deficiency_Code BY Anemia_History_Code
```

```
/STATISTICS=MEAN COUNT.
```

Means

Notes

Output Created		04-AUG-2025 13:19:46
Comments		
Input	Filter	<none>
	Weight	<none>
	Split File	<none>
	N of Rows in Working Data File	100
Missing Value Handling	Definition of Missing	For each dependent variable in a table, user-defined missing values for the dependent and all grouping variables are treated as missing.
	Cases Used	Cases used for each table have no missing values in any independent variable, and not all dependent variables have missing values.
Syntax		MEANS Iron_Deficiency_Code BY Anemia_History_Code /STATISTICS=MEAN COUNT.
Resources	Processor Time	00:00:00.02
	Elapsed Time	00:00:00.02

Warnings

Invalid Statistics subcommand. Found: MEAN
Invalid Statistics subcommand. Found: COUNT

Case Processing Summary

	Cases					
	Included		Excluded		Total	
	N	Percent	N	Percent	N	Percent
Iron Deficiency Status * History of Anemia	100	100.0%	0	0.0%	100	100.0%

Report

Iron Deficiency Status

History of Anemia	Mean	N	Std. Deviation
No	.8310	71	.37743
Yes	1.0000	29	.00000
Total	.8800	100	.32660

```
 ECHO "Analysis Complete - Iron Deficiency Study Data Generated Successfully".
Analysis Complete - Iron Deficiency Study Data Generated Successfully
* =======================================================
* FINAL CORRECTED IRON DEFICIENCY STUDY SYNTAX
* =======================================================

INPUT PROGRAM.
LOOP #I=1 TO 100.
COMPUTE Participant_ID=#I.


COMPUTE Age = 16 + TRUNC(UNIFORM(1) * 30).
COMPUTE BMI = NORMAL(22.5) + 3.5.
IF BMI < 16 BMI = 16.
IF BMI > 35 BMI = 35.
COMPUTE Hemoglobin_g_dL = NORMAL(12.5) + 0.8.
IF Hemoglobin_g_dL < 11.0 Hemoglobin_g_dL = 11.0.
IF Hemoglobin_g_dL > 15.0 Hemoglobin_g_dL = 15.0.
COMPUTE Serum_Ferritin_ug_L = EXP(NORMAL(2.8) + 1.0).
IF Serum_Ferritin_ug_L > 150 Serum_Ferritin_ug_L = 150.
COMPUTE Serum_Iron_umol_L = NORMAL(15) + 6.
IF Serum_Iron_umol_L < 5 Serum_Iron_umol_L = 5.
IF Serum_Iron_umol_L > 30 Serum_Iron_umol_L = 30.

COMPUTE Age_Group_Code = 2.
IF #I <= 34 Age_Group_Code = 1.
IF #I > 75 Age_Group_Code = 3.

COMPUTE BMI_Code = 2.
IF #I <= 15 BMI_Code = 1.
IF #I > 69 BMI_Code = 3.


COMPUTE Residence_Code = 2.
IF #I <= 38 Residence_Code = 1.

COMPUTE Marital_Code = 1.
IF #I <= 31 Marital_Code = 0.

COMPUTE Anemia_History_Code = 0.
IF #I <= 27 Anemia_History_Code = 1.

COMPUTE Hypertension_Code = 0.
IF #I <= 21 Hypertension_Code = 1.

COMPUTE Diabetes_Code = 0.
IF #I <= 13 Diabetes_Code = 1.

COMPUTE Breastfeeding_Code = 0.
IF #I <= 58 Breastfeeding_Code = 1.
```

```
IF #I <= 9 Smoking_Code = 1.

COMPUTE Contraceptive_Code = 0.
IF #I <= 36 Contraceptive_Code = 1.

COMPUTE Lifestyle_Code = 1.
IF #I <= 45 Lifestyle_Code = 0.

COMPUTE Number_of_Deliveries = 0.
IF Marital_Code = 1 AND UNIFORM(1) < 0.6 Number_of_Deliveries = TRUNC(UNIFORM(4) + 1).

COMPUTE Supplement_Code = 1.
IF UNIFORM(1) < 0.64 Supplement_Code = 0.

COMPUTE Meat_Intake_Code = 1.
IF UNIFORM(1) < 0.61 Meat_Intake_Code = 0.

COMPUTE Doctor_Visits_Code = 1.
IF UNIFORM(1) < 0.52 Doctor_Visits_Code = 0.

COMPUTE Iron_Deficiency_Code = 0.

IF #I <= 41 Iron_Deficiency_Code = 1.

IF BMI_Code = 1 AND Iron_Deficiency_Code = 0 AND UNIFORM(1) < 0.6 Iron_Deficiency_Code = 1.
IF Anemia_History_Code = 1 AND Iron_Deficiency_Code = 0 AND UNIFORM(1) < 0.5 Iron_Deficiency
IF Hypertension_Code = 1 AND Iron_Deficiency_Code = 0 AND UNIFORM(1) < 0.4 Iron_Deficiency_C


COMPUTE #total_positive = 0.
LOOP #j = 1 TO 100.
IF Iron_Deficiency_Code = 1 #total_positive = #total_positive + 1.
END LOOP.


IF #I <= 41 AND UNIFORM(1) < 0.8 Iron_Deficiency_Code = 1.
IF #I > 41 AND Iron_Deficiency_Code = 1 AND UNIFORM(1) < 0.4 Iron_Deficiency_Code = 0.

END CASE.
END LOOP.
END FILE.
END INPUT PROGRAM.

EXECUTE.

VARIABLE LABELS
  Participant_ID 'Participant ID'
  Age 'Age in years'
  BMI 'Body Mass Index (kg/m²)'
  Hemoglobin_g_dL 'Hemoglobin level (g/dL)'
  Serum_Ferritin_ug_L 'Serum Ferritin (µg/L)'
  Serum_Iron_umol_L 'Serum Iron (µmol/L)'
  Age_Group_Code 'Age Group'
```

```
  BMI_Code 'BMI Category'
  Residence_Code 'Residence'
  Marital_Code 'Marital Status'
  Anemia_History_Code 'History of Anemia'
  Hypertension_Code 'Hypertension'
  Diabetes_Code 'Diabetes'
  Breastfeeding_Code 'Breastfeeding History'
  Smoking_Code 'Smoking History'
  Contraceptive_Code 'Contraceptive Use'
  Lifestyle_Code 'Lifestyle'
  Number_of_Deliveries 'Number of deliveries'
  Supplement_Code 'Iron Supplement Use'
  Meat_Intake_Code 'Meat Intake Frequency'
  Doctor_Visits_Code 'Doctor Visit Frequency'
  Iron_Deficiency_Code 'Iron Deficiency Status'.

VALUE LABELS
  Age_Group_Code 1 '16-25 years' 2 '26-35 years' 3 '36-45 years'
  /BMI_Code 1 'Underweight' 2 'Normal' 3 'Overweight/Obese'
  /Residence_Code 1 'Rural' 2 'Urban'
  /Marital_Code 0 'Unmarried' 1 'Married'
  /Anemia_History_Code 0 'No' 1 'Yes'
  /Hypertension_Code 0 'No' 1 'Yes'
  /Diabetes_Code 0 'No' 1 'Yes'
  /Breastfeeding_Code 0 'No' 1 'Yes'
  /Smoking_Code 0 'No' 1 'Yes'
  /Contraceptive_Code 0 'No' 1 'Yes'
  /Lifestyle_Code 0 'Active' 1 'Sedentary'
  /Supplement_Code 0 'No' 1 'Yes'
  /Meat_Intake_Code 0 '<3 times/week' 1 '?3 times/week'
  /Doctor_Visits_Code 0 '<1/month' 1 '?1/month'
  /Iron_Deficiency_Code 0 'Negative' 1 'Positive'.

FREQUENCIES VARIABLES=BMI_Code Anemia_History_Code Hypertension_Code Iron_Deficiency_Code
  /ORDER=ANALYSIS.
```

Frequencies

Notes

Output Created		04-AUG-2025 13:28:38
Comments		
Input	Filter	<none>
	Weight	<none>
	Split File	<none>
	N of Rows in Working Data File	100
Missing Value Handling	Definition of Missing	User-defined missing values are treated as missing.
	Cases Used	Statistics are based on all cases with valid data.
Syntax		FREQUENCIES VARIABLES=BMI_Code Anemia_History_Code Hypertension_Code Iron_Deficiency_Code /ORDER=ANALYSIS.
Resources	Processor Time	00:00:00.02
	Elapsed Time	00:00:00.04

Statistics

		BMI Category	History of Anemia	Hypertension	Iron Deficiency Status
N	Valid	100	100	100	100
	Missing	0	0	0	0

Frequency Table

BMI Category

		Frequency	Percent	Valid Percent	Cumulative Percent
Valid	Underweight	15	15.0	15.0	15.0
	Normal	54	54.0	54.0	69.0
	Overweight/Obese	31	31.0	31.0	100.0
	Total	100	100.0	100.0	

History of Anemia

		Frequency	Percent	Valid Percent	Cumulative Percent
Valid	No	73	73.0	73.0	73.0
	Yes	27	27.0	27.0	100.0
	Total	100	100.0	100.0	

Hypertension

		Frequency	Percent	Valid Percent	Cumulative Percent
Valid	No	79	79.0	79.0	79.0
	Yes	21	21.0	21.0	100.0
	Total	100	100.0	100.0	

Iron Deficiency Status

		Frequency	Percent	Valid Percent	Cumulative Percent
Valid	Negative	59	59.0	59.0	59.0
	Positive	41	41.0	41.0	100.0
	Total	100	100.0	100.0	

```
CROSSTABS
 /TABLES=BMI_Code Anemia_History_Code Hypertension_Code BY Iron_Deficiency_Code
 /FORMAT=AVALUE TABLES
 /STATISTICS=CHISQ RISK PHI
 /CELLS=COUNT ROW TOTAL.
```

Crosstabs

Notes

Output Created		04-AUG-2025 13:28:38
Comments		
Input	Filter	<none>
	Weight	<none>
	Split File	<none>
	N of Rows in Working Data File	100
Missing Value Handling	Definition of Missing	User-defined missing values are treated as missing.
	Cases Used	Statistics for each table are based on all the cases with valid data in the specified range(s) for all variables in each table.
Syntax		CROSSTABS /TABLES=BMI_Code Anemia_History_Code Hypertension_Code BY Iron_Deficiency_Code /FORMAT=AVALUE TABLES /STATISTICS=CHISQ RISK PHI /CELLS=COUNT ROW TOTAL.

Notes

Resources	Processor Time	00:00:00.02
	Elapsed Time	00:00:00.03
	Dimensions Requested	2
	Cells Available	174762

Case Processing Summary

	Cases					
	Valid		Missing		Total	
	N	Percent	N	Percent	N	Percent
BMI Category * Iron Deficiency Status	100	100.0%	0	0.0%	100	100.0%
History of Anemia * Iron Deficiency Status	100	100.0%	0	0.0%	100	100.0%
Hypertension * Iron Deficiency Status	100	100.0%	0	0.0%	100	100.0%

BMI Category * Iron Deficiency Status

Crosstab

			Iron Deficiency Status		Total
			Negative	Positive	
BMI Category	Underweight	Count	0	15	15
		% within BMI Category	0.0%	100.0%	100.0%
		% of Total	0.0%	15.0%	15.0%
	Normal	Count	28	26	54
		% within BMI Category	51.9%	48.1%	100.0%
		% of Total	28.0%	26.0%	54.0%
	Overweight/Obese	Count	31	0	31
		% within BMI Category	100.0%	0.0%	100.0%
		% of Total	31.0%	0.0%	31.0%
Total		Count	59	41	100
		% within BMI Category	59.0%	41.0%	100.0%
		% of Total	59.0%	41.0%	100.0%

Chi-Square Tests

	Value	df	Asymp. Sig. (2-sided)
Pearson Chi-Square	44.268[a]	2	.000
Likelihood Ratio	60.586	2	.000
Linear-by-Linear Association	43.793	1	.000
N of Valid Cases	100		

a. 0 cells (0.0%) have expected count less than 5. The minimum expected count is 6.15.

Symmetric Measures

		Value	Approx. Sig.
Nominal by Nominal	Phi	.665	.000
	Cramer's V	.665	.000
N of Valid Cases		100	

a. Not assuming the null hypothesis.

b. Using the asymptotic standard error assuming the null hypothesis.

Risk Estimate

	Value
Odds Ratio for BMI Category (Underweight / Normal)	

a. Risk Estimate statistics cannot be computed. They are only computed for a 2*2 table without empty cells.

History of Anemia * Iron Deficiency Status

Crosstab

			Iron Deficiency Status		
			Negative	Positive	Total
History of Anemia	No	Count	59	14	73
		% within History of Anemia	80.8%	19.2%	100.0%
		% of Total	59.0%	14.0%	73.0%
	Yes	Count	0	27	27
		% within History of Anemia	0.0%	100.0%	100.0%
		% of Total	0.0%	27.0%	27.0%
Total		Count	59	41	100
		% within History of Anemia	59.0%	41.0%	100.0%
		% of Total	59.0%	41.0%	100.0%

Chi-Square Tests

	Value	df	Asymp. Sig. (2-sided)	Exact Sig. (2-sided)	Exact Sig. (1-sided)
Pearson Chi-Square	53.224[a]	1	.000		
Continuity Correction[b]	49.935	1	.000		
Likelihood Ratio	64.008	1	.000		
Fisher's Exact Test				.000	.000
Linear-by-Linear Association	52.692	1	.000		
N of Valid Cases	100				

a. 0 cells (0.0%) have expected count less than 5. The minimum expected count is 11.07.

b. Computed only for a 2x2 table

Symmetric Measures

		Value	Approx. Sig.
Nominal by Nominal	Phi	.730	.000
	Cramer's V	.730	.000
N of Valid Cases		100	

a. Not assuming the null hypothesis.

b. Using the asymptotic standard error assuming the null hypothesis.

Risk Estimate

	Value	95% Confidence Interval	
		Lower	Upper
For cohort Iron Deficiency Status = Positive	.192	.120	.307
N of Valid Cases	100		

Hypertension * Iron Deficiency Status

Crosstab

			Iron Deficiency Status		Total
			Negative	Positive	
Hypertension	No	Count	59	20	79
		% within Hypertension	74.7%	25.3%	100.0%
		% of Total	59.0%	20.0%	79.0%
	Yes	Count	0	21	21
		% within Hypertension	0.0%	100.0%	100.0%
		% of Total	0.0%	21.0%	21.0%
Total		Count	59	41	100
		% within Hypertension	59.0%	41.0%	100.0%
		% of Total	59.0%	41.0%	100.0%

Chi-Square Tests

	Value	df	Asymp. Sig. (2-sided)	Exact Sig. (2-sided)	Exact Sig. (1-sided)
Pearson Chi-Square	38.253[a]	1	.000		
Continuity Correction[b]	35.227	1	.000		
Likelihood Ratio	45.978	1	.000		
Fisher's Exact Test				.000	.000
Linear-by-Linear Association	37.870	1	.000		
N of Valid Cases	100				

a. 0 cells (0.0%) have expected count less than 5. The minimum expected count is 8.61.

b. Computed only for a 2x2 table

Symmetric Measures

		Value	Approx. Sig.
Nominal by Nominal	Phi	.618	.000
	Cramer's V	.618	.000
N of Valid Cases		100	

a. Not assuming the null hypothesis.

b. Using the asymptotic standard error assuming the null hypothesis.

Risk Estimate

	Value	95% Confidence Interval	
		Lower	Upper
For cohort Iron Deficiency Status = Positive	.253	.173	.370
N of Valid Cases	100		

```
 ECHO "FINAL Analysis Complete - All warnings resolved".
FINAL Analysis Complete - All warnings resolved
* =======================================================
* FINAL CORRECTED IRON DEFICIENCY STUDY - NO WARNINGS
* =======================================================

INPUT PROGRAM.
LOOP #I=1 TO 100.
COMPUTE Participant_ID=#I.


COMPUTE Age = 16 + TRUNC(UNIFORM(1) * 30).
COMPUTE BMI = NORMAL(22.5) + 3.5.
IF BMI < 16 BMI = 16.
IF BMI > 35 BMI = 35.
COMPUTE Hemoglobin_g_dL = NORMAL(12.5) + 0.8.
```

```
IF Hemoglobin_g_dL < 11.0 Hemoglobin_g_dL = 11.0.
IF Hemoglobin_g_dL > 15.0 Hemoglobin_g_dL = 15.0.


COMPUTE Serum_Ferritin_ug_L = EXP(NORMAL(2.5) + 1.2).
IF Serum_Ferritin_ug_L > 150 Serum_Ferritin_ug_L = 150.
COMPUTE Serum_Iron_umol_L = NORMAL(13) + 7.
IF Serum_Iron_umol_L < 5 Serum_Iron_umol_L = 5.
IF Serum_Iron_umol_L > 30 Serum_Iron_umol_L = 30.

COMPUTE Age_Group_Code = 2.
IF #I <= 34 Age_Group_Code = 1.
IF #I > 75 Age_Group_Code = 3.


COMPUTE BMI_Code = 2.
IF #I <= 15 BMI_Code = 1.
IF #I > 69 BMI_Code = 3.


COMPUTE Education_Code = 2.
IF #I <= 26 Education_Code = 1.
IF #I > 68 Education_Code = 3.


COMPUTE Residence_Code = 2.
IF #I <= 38 Residence_Code = 1.

COMPUTE Marital_Code = 1.
IF #I <= 31 Marital_Code = 0.

COMPUTE Anemia_History_Code = 0.
IF #I <= 27 Anemia_History_Code = 1.

COMPUTE Hypertension_Code = 0.
IF #I <= 21 Hypertension_Code = 1.

COMPUTE Diabetes_Code = 0.
IF #I <= 13 Diabetes_Code = 1.

COMPUTE Breastfeeding_Code = 0.
IF #I <= 58 Breastfeeding_Code = 1.

COMPUTE Smoking_Code = 0.
IF #I <= 9 Smoking_Code = 1.

COMPUTE Contraceptive_Code = 0.
IF #I <= 36 Contraceptive_Code = 1.

COMPUTE Lifestyle_Code = 1.
IF #I <= 45 Lifestyle_Code = 0.

COMPUTE Number_of_Deliveries = 0.
```

```
IF Marital_Code = 1 AND UNIFORM(1) < 0.6 Number_of_Deliveries = TRUNC(UNIFORM(4) + 1).

COMPUTE Supplement_Code = 1.
IF #I <= 64 Supplement_Code = 0.

COMPUTE Meat_Intake_Code = 1.
IF #I <= 61 Meat_Intake_Code = 0.

COMPUTE Doctor_Visits_Code = 1.
IF #I <= 52 Doctor_Visits_Code = 0.


COMPUTE Iron_Deficiency_Code = 0.


IF #I <= 41 Iron_Deficiency_Code = 1.


COMPUTE #risk_score = 0.


IF BMI_Code = 1 #risk_score = #risk_score + 3.
IF Anemia_History_Code = 1 #risk_score = #risk_score + 2.
IF Hypertension_Code = 1 #risk_score = #risk_score + 1.
IF Breastfeeding_Code = 1 #risk_score = #risk_score + 1.
IF Lifestyle_Code = 1 #risk_score = #risk_score + 1.


IF #I > 41 AND #risk_score >= 4 AND UNIFORM(1) < 0.7 Iron_Deficiency_Code = 1.
IF #I <= 41 AND #risk_score = 0 AND UNIFORM(1) < 0.3 Iron_Deficiency_Code = 0.


COMPUTE #running_total = 0.
LOOP #j = 1 TO #I.

END LOOP.


IF #I <= 41 Iron_Deficiency_Code = 1.

END CASE.
END LOOP.
END FILE.
END INPUT PROGRAM.

EXECUTE.


VARIABLE LABELS
  Participant_ID 'Participant ID'
  Age 'Age in years'
  BMI 'Body Mass Index (kg/m²)'
  Hemoglobin_g_dL 'Hemoglobin level (g/dL)'
```

```
  Serum_Ferritin_ug_L 'Serum Ferritin (μg/L)'
  Serum_Iron_umol_L 'Serum Iron (μmol/L)'
  Age_Group_Code 'Age Group'
  Education_Code 'Education Level'
  BMI_Code 'BMI Category'
  Residence_Code 'Residence'
  Marital_Code 'Marital Status'
  Anemia_History_Code 'History of Anemia'
  Hypertension_Code 'Hypertension'
  Diabetes_Code 'Diabetes'
  Breastfeeding_Code 'Breastfeeding History'
  Smoking_Code 'Smoking History'
  Contraceptive_Code 'Contraceptive Use'
  Lifestyle_Code 'Lifestyle'
  Number_of_Deliveries 'Number of deliveries'
  Supplement_Code 'Iron Supplement Use'
  Meat_Intake_Code 'Meat Intake Frequency'
  Doctor_Visits_Code 'Doctor Visit Frequency'
  Iron_Deficiency_Code 'Iron Deficiency Status'.

VALUE LABELS
  Age_Group_Code 1 '16-25 years' 2 '26-35 years' 3 '36-45 years'
  /Education_Code 1 'Below Secondary' 2 'Secondary' 3 'Graduate+'
  /BMI_Code 1 'Underweight' 2 'Normal' 3 'Overweight/Obese'
  /Residence_Code 1 'Rural' 2 'Urban'
  /Marital_Code 0 'Unmarried' 1 'Married'
  /Anemia_History_Code 0 'No' 1 'Yes'
  /Hypertension_Code 0 'No' 1 'Yes'
  /Diabetes_Code 0 'No' 1 'Yes'
  /Breastfeeding_Code 0 'No' 1 'Yes'
  /Smoking_Code 0 'No' 1 'Yes'
  /Contraceptive_Code 0 'No' 1 'Yes'
  /Lifestyle_Code 0 'Active' 1 'Sedentary'
  /Supplement_Code 0 'No' 1 'Yes'
  /Meat_Intake_Code 0 '<3 times/week' 1 '?3 times/week'
  /Doctor_Visits_Code 0 '<1/month' 1 '?1/month'
  /Iron_Deficiency_Code 0 'Negative' 1 'Positive'.

MISSING VALUES
  Age (99) BMI (99.9) Hemoglobin_g_dL (99.9) Serum_Ferritin_ug_L (999.9)
  Serum_Iron_umol_L (999.9) Number_of_Deliveries (9)
  Age_Group_Code Education_Code BMI_Code Residence_Code Marital_Code
  Anemia_History_Code Hypertension_Code Diabetes_Code Breastfeeding_Code
  Smoking_Code Contraceptive_Code Lifestyle_Code Supplement_Code
  Meat_Intake_Code Doctor_Visits_Code Iron_Deficiency_Code (9).


FREQUENCIES VARIABLES=Iron_Deficiency_Code BMI_Code Anemia_History_Code Hypertension_Code
  /ORDER=ANALYSIS.
```

Frequencies

Notes

Output Created		04-AUG-2025 13:36:03
Comments		
Input	Filter	<none>
	Weight	<none>
	Split File	<none>
	N of Rows in Working Data File	100
Missing Value Handling	Definition of Missing	User-defined missing values are treated as missing.
	Cases Used	Statistics are based on all cases with valid data.
Syntax		FREQUENCIES VARIABLES=Iron_Deficiency_Code BMI_Code Anemia_History_Code Hypertension_Code /ORDER=ANALYSIS.
Resources	Processor Time	00:00:00.02
	Elapsed Time	00:00:00.02

Statistics

		Iron Deficiency Status	BMI Category	History of Anemia	Hypertension
N	Valid	100	100	100	100
	Missing	0	0	0	0

Frequency Table

Iron Deficiency Status

		Frequency	Percent	Valid Percent	Cumulative Percent
Valid	Negative	59	59.0	59.0	59.0
	Positive	41	41.0	41.0	100.0
	Total	100	100.0	100.0	

BMI Category

		Frequency	Percent	Valid Percent	Cumulative Percent
Valid	Underweight	15	15.0	15.0	15.0
	Normal	54	54.0	54.0	69.0
	Overweight/Obese	31	31.0	31.0	100.0
	Total	100	100.0	100.0	

History of Anemia

		Frequency	Percent	Valid Percent	Cumulative Percent
Valid	No	73	73.0	73.0	73.0
	Yes	27	27.0	27.0	100.0
	Total	100	100.0	100.0	

Hypertension

		Frequency	Percent	Valid Percent	Cumulative Percent
Valid	No	79	79.0	79.0	79.0
	Yes	21	21.0	21.0	100.0
	Total	100	100.0	100.0	

```
CROSSTABS
  /TABLES=BMI_Code Anemia_History_Code Hypertension_Code BY Iron_Deficiency_Code
  /FORMAT=AVALUE TABLES
  /STATISTICS=CHISQ RISK PHI
  /CELLS=COUNT ROW TOTAL.
```

Crosstabs

Notes

Output Created		04-AUG-2025 13:36:03
Comments		
Input	Filter	<none>
	Weight	<none>
	Split File	<none>
	N of Rows in Working Data File	100
Missing Value Handling	Definition of Missing	User-defined missing values are treated as missing.
	Cases Used	Statistics for each table are based on all the cases with valid data in the specified range(s) for all variables in each table.
Syntax		CROSSTABS /TABLES=BMI_Code Anemia_History_Code Hypertension_Code BY Iron_Deficiency_Code /FORMAT=AVALUE TABLES /STATISTICS=CHISQ RISK PHI /CELLS=COUNT ROW TOTAL.

Notes

Resources	Processor Time	00:00:00.03
	Elapsed Time	00:00:00.05
	Dimensions Requested	2
	Cells Available	174762

Case Processing Summary

	Cases					
	Valid		Missing		Total	
	N	Percent	N	Percent	N	Percent
BMI Category * Iron Deficiency Status	100	100.0%	0	0.0%	100	100.0%
History of Anemia * Iron Deficiency Status	100	100.0%	0	0.0%	100	100.0%
Hypertension * Iron Deficiency Status	100	100.0%	0	0.0%	100	100.0%

BMI Category * Iron Deficiency Status

Crosstab

			Iron Deficiency Status		Total
			Negative	Positive	
BMI Category	Underweight	Count	0	15	15
		% within BMI Category	0.0%	100.0%	100.0%
		% of Total	0.0%	15.0%	15.0%
	Normal	Count	28	26	54
		% within BMI Category	51.9%	48.1%	100.0%
		% of Total	28.0%	26.0%	54.0%
	Overweight/Obese	Count	31	0	31
		% within BMI Category	100.0%	0.0%	100.0%
		% of Total	31.0%	0.0%	31.0%
Total		Count	59	41	100
		% within BMI Category	59.0%	41.0%	100.0%
		% of Total	59.0%	41.0%	100.0%

Chi-Square Tests

	Value	df	Asymp. Sig. (2-sided)
Pearson Chi-Square	44.268[a]	2	.000
Likelihood Ratio	60.586	2	.000
Linear-by-Linear Association	43.793	1	.000
N of Valid Cases	100		

a. 0 cells (0.0%) have expected count less than 5. The minimum expected count is 6.15.

Symmetric Measures

		Value	Approx. Sig.
Nominal by Nominal	Phi	.665	.000
	Cramer's V	.665	.000
N of Valid Cases		100	

a. Not assuming the null hypothesis.

b. Using the asymptotic standard error assuming the null hypothesis.

Risk Estimate

	Value
Odds Ratio for BMI Category (Underweight / Normal)	

a. Risk Estimate statistics cannot be computed. They are only computed for a 2*2 table without empty cells.

History of Anemia * Iron Deficiency Status

Crosstab

			Iron Deficiency Status		
			Negative	Positive	Total
History of Anemia	No	Count	59	14	73
		% within History of Anemia	80.8%	19.2%	100.0%
		% of Total	59.0%	14.0%	73.0%
	Yes	Count	0	27	27
		% within History of Anemia	0.0%	100.0%	100.0%
		% of Total	0.0%	27.0%	27.0%
Total		Count	59	41	100
		% within History of Anemia	59.0%	41.0%	100.0%
		% of Total	59.0%	41.0%	100.0%

Chi-Square Tests

	Value	df	Asymp. Sig. (2-sided)	Exact Sig. (2-sided)	Exact Sig. (1-sided)
Pearson Chi-Square	53.224[a]	1	.000		
Continuity Correction[b]	49.935	1	.000		
Likelihood Ratio	64.008	1	.000		
Fisher's Exact Test				.000	.000
Linear-by-Linear Association	52.692	1	.000		
N of Valid Cases	100				

a. 0 cells (0.0%) have expected count less than 5. The minimum expected count is 11.07.

b. Computed only for a 2x2 table

Symmetric Measures

		Value	Approx. Sig.
Nominal by Nominal	Phi	.730	.000
	Cramer's V	.730	.000
N of Valid Cases		100	

a. Not assuming the null hypothesis.

b. Using the asymptotic standard error assuming the null hypothesis.

Risk Estimate

	Value	95% Confidence Interval	
		Lower	Upper
For cohort Iron Deficiency Status = Positive	.192	.120	.307
N of Valid Cases	100		

Hypertension * Iron Deficiency Status

Crosstab

			Iron Deficiency Status		Total
			Negative	Positive	
Hypertension	No	Count	59	20	79
		% within Hypertension	74.7%	25.3%	100.0%
		% of Total	59.0%	20.0%	79.0%
	Yes	Count	0	21	21
		% within Hypertension	0.0%	100.0%	100.0%
		% of Total	0.0%	21.0%	21.0%
Total		Count	59	41	100
		% within Hypertension	59.0%	41.0%	100.0%
		% of Total	59.0%	41.0%	100.0%

Chi-Square Tests

	Value	df	Asymp. Sig. (2-sided)	Exact Sig. (2-sided)	Exact Sig. (1-sided)
Pearson Chi-Square	38.253[a]	1	.000		
Continuity Correction[b]	35.227	1	.000		
Likelihood Ratio	45.978	1	.000		
Fisher's Exact Test				.000	.000
Linear-by-Linear Association	37.870	1	.000		
N of Valid Cases	100				

a. 0 cells (0.0%) have expected count less than 5. The minimum expected count is 8.61.

b. Computed only for a 2x2 table

Symmetric Measures

		Value	Approx. Sig.
Nominal by Nominal	Phi	.618	.000
	Cramer's V	.618	.000
N of Valid Cases		100	

a. Not assuming the null hypothesis.

b. Using the asymptotic standard error assuming the null hypothesis.

Risk Estimate

	Value	95% Confidence Interval	
		Lower	Upper
For cohort Iron Deficiency Status = Positive	.253	.173	.370
N of Valid Cases	100		

```
LOGISTIC REGRESSION VARIABLES Iron_Deficiency_Code
 /METHOD=ENTER BMI_Code Anemia_History_Code Hypertension_Code
   Breastfeeding_Code Lifestyle_Code Supplement_Code Meat_Intake_Code
 /CRITERIA=PIN(0.05) POUT(0.10) ITERATE(20) CUT(0.5).
```

Logistic Regression

Notes

Output Created		04-AUG-2025 13:36:03
Comments		
Input	Filter	<none>
	Weight	<none>
	Split File	<none>
	N of Rows in Working Data File	100
Missing Value Handling	Definition of Missing	User-defined missing values are treated as missing
Syntax		LOGISTIC REGRESSION VARIABLES Iron_Deficiency_Code /METHOD=ENTER BMI_Code Anemia_History_Code Hypertension_Code Breastfeeding_Code Lifestyle_Code Supplement_Code Meat_Intake_Code /CRITERIA=PIN(0.05) POUT(0.10) ITERATE(20) CUT(0.5).
Resources	Processor Time	00:00:00.03
	Elapsed Time	00:00:00.06

Case Processing Summary

Unweighted Cases[a]		N	Percent
Selected Cases	Included in Analysis	100	100.0
	Missing Cases	0	.0
	Total	100	100.0
Unselected Cases		0	.0
Total		100	100.0

a. If weight is in effect, see classification table for the total number of cases.

Dependent Variable Encoding

Original Value	Internal Value
Negative	0
Positive	1

Block 0: Beginning Block

Classification Table[a,b]

			Predicted		
			Iron Deficiency Status		Percentage Correct
Observed			Negative	Positive	
Step 0	Iron Deficiency Status	Negative	59	0	100.0
		Positive	41	0	.0
	Overall Percentage				59.0

a. Constant is included in the model.

b. The cut value is .500

Variables in the Equation

		B	S.E.	Wald	df	Sig.	Exp(B)
Step 0	Constant	-.364	.203	3.204	1	.073	.695

Variables not in the Equation

			Score	df	Sig.
Step 0	Variables	BMI_Code	44.236	1	.000
		Anemia_History_Code	53.224	1	.000
		Hypertension_Code	38.253	1	.000
		Breastfeeding_Code	50.321	1	.000
		Lifestyle_Code	84.934	1	.000
		Supplement_Code	39.089	1	.000
		Meat_Intake_Code	44.429	1	.000
	Overall Statistics		87.139	7	.000

Block 1: Method = Enter

Omnibus Tests of Model Coefficients

		Chi-square	df	Sig.
Step 1	Step	116.302	7	.000
	Block	116.302	7	.000
	Model	116.302	7	.000

Model Summary

Step	-2 Log likelihood	Cox & Snell R Square	Nagelkerke R Square
1	19.069[a]	.687	.927

a. Estimation terminated at iteration number 20 because maximum iterations has been reached. Final solution cannot be found.

Classification Table[a]

Observed			Predicted		
			Iron Deficiency Status		Percentage Correct
			Negative	Positive	
Step 1	Iron Deficiency Status	Negative	55	4	93.2
		Positive	0	41	100.0
	Overall Percentage				96.0

a. The cut value is .500

Variables in the Equation

		B	S.E.	Wald	df	Sig.
Step 1[a]	BMI_Code	.000	13712.680	.000	1	1.000
	Anemia_History_Code	19.950	16408.711	.000	1	.999
	Hypertension_Code	.000	21026.429	.000	1	1.000
	Breastfeeding_Code	.000	25744.177	.000	1	1.000
	Lifestyle_Code	-22.456	11147.530	.000	1	.998
	Supplement_Code	.000	26884.905	.000	1	1.000
	Meat_Intake_Code	.000	32817.460	.000	1	1.000
	Constant	1.253	37615.380	.000	1	1.000

Variables in the Equation

		Exp(B)
Step 1[a]	BMI_Code	1.000
	Anemia_History_Code	461564247.1
	Hypertension_Code	1.000
	Breastfeeding_Code	1.000
	Lifestyle_Code	.000
	Supplement_Code	1.000
	Meat_Intake_Code	1.000
	Constant	3.500

a. Variable(s) entered on step 1: BMI_Code, Anemia_History_Code, Hypertension_Code, Breastfeeding_Code, Lifestyle_Code, Supplement_Code, Meat_Intake_Code.

```
INPUT PROGRAM.
LOOP #I=1 TO 100.
COMPUTE Participant_ID=#I.
```

```
COMPUTE Age = 16 + TRUNC(UNIFORM(1) * 30).
COMPUTE BMI = NORMAL(22.5) + 3.5.
IF BMI < 16 BMI = 16.
IF BMI > 35 BMI = 35.
COMPUTE Hemoglobin_g_dL = NORMAL(12.5) + 0.8.
IF Hemoglobin_g_dL < 11.0 Hemoglobin_g_dL = 11.0.
IF Hemoglobin_g_dL > 15.0 Hemoglobin_g_dL = 15.0.


COMPUTE Serum_Ferritin_ug_L = EXP(NORMAL(2.5) + 1.2).
IF Serum_Ferritin_ug_L > 150 Serum_Ferritin_ug_L = 150.
COMPUTE Serum_Iron_umol_L = NORMAL(13) + 7.
IF Serum_Iron_umol_L < 5 Serum_Iron_umol_L = 5.
IF Serum_Iron_umol_L > 30 Serum_Iron_umol_L = 30.

COMPUTE Age_Group_Code = 2.
IF #I <= 34 Age_Group_Code = 1.
IF #I > 75 Age_Group_Code = 3.


COMPUTE BMI_Code = 2.
IF #I <= 15 BMI_Code = 1.
IF #I > 69 BMI_Code = 3.


COMPUTE Education_Code = 2.
IF #I <= 26 Education_Code = 1.
IF #I > 68 Education_Code = 3.


COMPUTE Residence_Code = 2.
IF #I <= 38 Residence_Code = 1.

COMPUTE Marital_Code = 1.
IF #I <= 31 Marital_Code = 0.

COMPUTE Anemia_History_Code = 0.
IF #I <= 27 Anemia_History_Code = 1.

COMPUTE Hypertension_Code = 0.
IF #I <= 21 Hypertension_Code = 1.

COMPUTE Diabetes_Code = 0.
IF #I <= 13 Diabetes_Code = 1.

COMPUTE Breastfeeding_Code = 0.
IF #I <= 58 Breastfeeding_Code = 1.

COMPUTE Smoking_Code = 0.
IF #I <= 9 Smoking_Code = 1.

COMPUTE Contraceptive_Code = 0.
```

```
IF #I <= 36 Contraceptive_Code = 1.

COMPUTE Lifestyle_Code = 1.
IF #I <= 45 Lifestyle_Code = 0.

COMPUTE Number_of_Deliveries = 0.
IF Marital_Code = 1 AND UNIFORM(1) < 0.6 Number_of_Deliveries = TRUNC(UNIFORM(4) + 1).

COMPUTE Supplement_Code = 1.
IF #I <= 64 Supplement_Code = 0.

COMPUTE Meat_Intake_Code = 1.
IF #I <= 61 Meat_Intake_Code = 0.

COMPUTE Doctor_Visits_Code = 1.
IF #I <= 52 Doctor_Visits_Code = 0.


COMPUTE Iron_Deficiency_Code = 0.


IF #I <= 41 Iron_Deficiency_Code = 1.


COMPUTE #risk_score = 0.


IF BMI_Code = 1 #risk_score = #risk_score + 3.
IF Anemia_History_Code = 1 #risk_score = #risk_score + 2.
IF Hypertension_Code = 1 #risk_score = #risk_score + 1.
IF Breastfeeding_Code = 1 #risk_score = #risk_score + 1.
IF Lifestyle_Code = 1 #risk_score = #risk_score + 1.


IF #I > 41 AND #risk_score >= 4 AND UNIFORM(1) < 0.7 Iron_Deficiency_Code = 1.
IF #I <= 41 AND #risk_score = 0 AND UNIFORM(1) < 0.3 Iron_Deficiency_Code = 0.


COMPUTE #running_total = 0.
LOOP #j = 1 TO #I.

END LOOP.


IF #I <= 41 Iron_Deficiency_Code = 1.

END CASE.
END LOOP.
END FILE.
END INPUT PROGRAM.

EXECUTE.
```

```
VARIABLE LABELS
  Participant_ID 'Participant ID'
  Age 'Age in years'
  BMI 'Body Mass Index (kg/m²)'
  Hemoglobin_g_dL 'Hemoglobin level (g/dL)'
  Serum_Ferritin_ug_L 'Serum Ferritin (µg/L)'
  Serum_Iron_umol_L 'Serum Iron (µmol/L)'
  Age_Group_Code 'Age Group'
  Education_Code 'Education Level'
  BMI_Code 'BMI Category'
  Residence_Code 'Residence'
  Marital_Code 'Marital Status'
  Anemia_History_Code 'History of Anemia'
  Hypertension_Code 'Hypertension'
  Diabetes_Code 'Diabetes'
  Breastfeeding_Code 'Breastfeeding History'
  Smoking_Code 'Smoking History'
  Contraceptive_Code 'Contraceptive Use'
  Lifestyle_Code 'Lifestyle'
  Number_of_Deliveries 'Number of deliveries'
  Supplement_Code 'Iron Supplement Use'
  Meat_Intake_Code 'Meat Intake Frequency'
  Doctor_Visits_Code 'Doctor Visit Frequency'
  Iron_Deficiency_Code 'Iron Deficiency Status'.

VALUE LABELS
  Age_Group_Code 1 '16-25 years' 2 '26-35 years' 3 '36-45 years'
  /Education_Code 1 'Below Secondary' 2 'Secondary' 3 'Graduate+'
  /BMI_Code 1 'Underweight' 2 'Normal' 3 'Overweight/Obese'
  /Residence_Code 1 'Rural' 2 'Urban'
  /Marital_Code 0 'Unmarried' 1 'Married'
  /Anemia_History_Code 0 'No' 1 'Yes'
  /Hypertension_Code 0 'No' 1 'Yes'
  /Diabetes_Code 0 'No' 1 'Yes'
  /Breastfeeding_Code 0 'No' 1 'Yes'
  /Smoking_Code 0 'No' 1 'Yes'
  /Contraceptive_Code 0 'No' 1 'Yes'
  /Lifestyle_Code 0 'Active' 1 'Sedentary'
  /Supplement_Code 0 'No' 1 'Yes'
  /Meat_Intake_Code 0 '<3 times/week' 1 '?3 times/week'
  /Doctor_Visits_Code 0 '<1/month' 1 '?1/month'
  /Iron_Deficiency_Code 0 'Negative' 1 'Positive'.

MISSING VALUES
  Age (99) BMI (99.9) Hemoglobin_g_dL (99.9) Serum_Ferritin_ug_L (999.9)
  Serum_Iron_umol_L (999.9) Number_of_Deliveries (9)
  Age_Group_Code Education_Code BMI_Code Residence_Code Marital_Code
  Anemia_History_Code Hypertension_Code Diabetes_Code Breastfeeding_Code
  Smoking_Code Contraceptive_Code Lifestyle_Code Supplement_Code
  Meat_Intake_Code Doctor_Visits_Code Iron_Deficiency_Code (9).
```

```
FREQUENCIES VARIABLES=Iron_Deficiency_Code BMI_Code Anemia_History_Code Hypertension_Code
  /ORDER=ANALYSIS.
```

Frequencies

Notes

Output Created		04-AUG-2025 13:41:06
Comments		
Input	Filter	<none>
	Weight	<none>
	Split File	<none>
	N of Rows in Working Data File	100
Missing Value Handling	Definition of Missing	User-defined missing values are treated as missing.
	Cases Used	Statistics are based on all cases with valid data.
Syntax		FREQUENCIES VARIABLES=Iron_Deficiency_Code BMI_Code Anemia_History_Code Hypertension_Code /ORDER=ANALYSIS.
Resources	Processor Time	00:00:00.03
	Elapsed Time	00:00:00.01

Statistics

		Iron Deficiency Status	BMI Category	History of Anemia	Hypertension
N	Valid	100	100	100	100
	Missing	0	0	0	0

Frequency Table

Iron Deficiency Status

		Frequency	Percent	Valid Percent	Cumulative Percent
Valid	Negative	59	59.0	59.0	59.0
	Positive	41	41.0	41.0	100.0
	Total	100	100.0	100.0	

BMI Category

		Frequency	Percent	Valid Percent	Cumulative Percent
Valid	Underweight	15	15.0	15.0	15.0
	Normal	54	54.0	54.0	69.0
	Overweight/Obese	31	31.0	31.0	100.0
	Total	100	100.0	100.0	

History of Anemia

		Frequency	Percent	Valid Percent	Cumulative Percent
Valid	No	73	73.0	73.0	73.0
	Yes	27	27.0	27.0	100.0
	Total	100	100.0	100.0	

Hypertension

		Frequency	Percent	Valid Percent	Cumulative Percent
Valid	No	79	79.0	79.0	79.0
	Yes	21	21.0	21.0	100.0
	Total	100	100.0	100.0	

```
CROSSTABS
 /TABLES=BMI_Code Anemia_History_Code Hypertension_Code BY Iron_Deficiency_Code
 /FORMAT=AVALUE TABLES
 /STATISTICS=CHISQ RISK PHI
 /CELLS=COUNT ROW TOTAL.
```

Crosstabs

Notes

Output Created		04-AUG-2025 13:41:06
Comments		
Input	Filter	<none>
	Weight	<none>
	Split File	<none>
	N of Rows in Working Data File	100
Missing Value Handling	Definition of Missing	User-defined missing values are treated as missing.
	Cases Used	Statistics for each table are based on all the cases with valid data in the specified range(s) for all variables in each table.
Syntax		CROSSTABS /TABLES=BMI_Code Anemia_History_Code Hypertension_Code BY Iron_Deficiency_Code /FORMAT=AVALUE TABLES /STATISTICS=CHISQ RISK PHI /CELLS=COUNT ROW TOTAL.

Notes

Resources	Processor Time	00:00:00.02
	Elapsed Time	00:00:00.02
	Dimensions Requested	2
	Cells Available	174762

Case Processing Summary

	Cases					
	Valid		Missing		Total	
	N	Percent	N	Percent	N	Percent
BMI Category * Iron Deficiency Status	100	100.0%	0	0.0%	100	100.0%
History of Anemia * Iron Deficiency Status	100	100.0%	0	0.0%	100	100.0%
Hypertension * Iron Deficiency Status	100	100.0%	0	0.0%	100	100.0%

BMI Category * Iron Deficiency Status

Crosstab

			Iron Deficiency Status		
			Negative	Positive	Total
BMI Category	Underweight	Count	0	15	15
		% within BMI Category	0.0%	100.0%	100.0%
		% of Total	0.0%	15.0%	15.0%
	Normal	Count	28	26	54
		% within BMI Category	51.9%	48.1%	100.0%
		% of Total	28.0%	26.0%	54.0%
	Overweight/Obese	Count	31	0	31
		% within BMI Category	100.0%	0.0%	100.0%
		% of Total	31.0%	0.0%	31.0%
Total		Count	59	41	100
		% within BMI Category	59.0%	41.0%	100.0%
		% of Total	59.0%	41.0%	100.0%

Chi-Square Tests

	Value	df	Asymp. Sig. (2-sided)
Pearson Chi-Square	44.268[a]	2	.000
Likelihood Ratio	60.586	2	.000
Linear-by-Linear Association	43.793	1	.000
N of Valid Cases	100		

a. 0 cells (0.0%) have expected count less than 5. The minimum expected count is 6.15.

Symmetric Measures

		Value	Approx. Sig.
Nominal by Nominal	Phi	.665	.000
	Cramer's V	.665	.000
N of Valid Cases		100	

a. Not assuming the null hypothesis.

b. Using the asymptotic standard error assuming the null hypothesis.

Risk Estimate

	Value
Odds Ratio for BMI Category (Underweight / Normal)	

a. Risk Estimate statistics cannot be computed. They are only computed for a 2*2 table without empty cells.

History of Anemia * Iron Deficiency Status

Crosstab

			Iron Deficiency Status		
			Negative	Positive	Total
History of Anemia	No	Count	59	14	73
		% within History of Anemia	80.8%	19.2%	100.0%
		% of Total	59.0%	14.0%	73.0%
	Yes	Count	0	27	27
		% within History of Anemia	0.0%	100.0%	100.0%
		% of Total	0.0%	27.0%	27.0%
Total		Count	59	41	100
		% within History of Anemia	59.0%	41.0%	100.0%
		% of Total	59.0%	41.0%	100.0%

Chi-Square Tests

	Value	df	Asymp. Sig. (2-sided)	Exact Sig. (2-sided)	Exact Sig. (1-sided)
Pearson Chi-Square	53.224[a]	1	.000		
Continuity Correction[b]	49.935	1	.000		
Likelihood Ratio	64.008	1	.000		
Fisher's Exact Test				.000	.000
Linear-by-Linear Association	52.692	1	.000		
N of Valid Cases	100				

a. 0 cells (0.0%) have expected count less than 5. The minimum expected count is 11.07.

b. Computed only for a 2x2 table

Symmetric Measures

		Value	Approx. Sig.
Nominal by Nominal	Phi	.730	.000
	Cramer's V	.730	.000
N of Valid Cases		100	

a. Not assuming the null hypothesis.

b. Using the asymptotic standard error assuming the null hypothesis.

Risk Estimate

	Value	95% Confidence Interval	
		Lower	Upper
For cohort Iron Deficiency Status = Positive	.192	.120	.307
N of Valid Cases	100		

Hypertension * Iron Deficiency Status

Crosstab

			Iron Deficiency Status		Total
			Negative	Positive	
Hypertension	No	Count	59	20	79
		% within Hypertension	74.7%	25.3%	100.0%
		% of Total	59.0%	20.0%	79.0%
	Yes	Count	0	21	21
		% within Hypertension	0.0%	100.0%	100.0%
		% of Total	0.0%	21.0%	21.0%
Total		Count	59	41	100
		% within Hypertension	59.0%	41.0%	100.0%
		% of Total	59.0%	41.0%	100.0%

Chi-Square Tests

	Value	df	Asymp. Sig. (2-sided)	Exact Sig. (2-sided)	Exact Sig. (1-sided)
Pearson Chi-Square	38.253[a]	1	.000		
Continuity Correction[b]	35.227	1	.000		
Likelihood Ratio	45.978	1	.000		
Fisher's Exact Test				.000	.000
Linear-by-Linear Association	37.870	1	.000		
N of Valid Cases	100				

a. 0 cells (0.0%) have expected count less than 5. The minimum expected count is 8.61.

b. Computed only for a 2x2 table

Symmetric Measures

		Value	Approx. Sig.
Nominal by Nominal	Phi	.618	.000
	Cramer's V	.618	.000
N of Valid Cases		100	

a. Not assuming the null hypothesis.

b. Using the asymptotic standard error assuming the null hypothesis.

Risk Estimate

	Value	95% Confidence Interval	
		Lower	Upper
For cohort Iron Deficiency Status = Positive	.253	.173	.370
N of Valid Cases	100		

```
LOGISTIC REGRESSION VARIABLES Iron_Deficiency_Code
 /METHOD=ENTER BMI_Code Anemia_History_Code Hypertension_Code
   Breastfeeding_Code Lifestyle_Code Supplement_Code Meat_Intake_Code
 /CRITERIA=PIN(0.05) POUT(0.10) ITERATE(20) CUT(0.5).
```

Logistic Regression

Notes

Output Created		04-AUG-2025 13:41:06
Comments		
Input	Filter	<none>
	Weight	<none>
	Split File	<none>
	N of Rows in Working Data File	100
Missing Value Handling	Definition of Missing	User-defined missing values are treated as missing
Syntax		LOGISTIC REGRESSION VARIABLES Iron_Deficiency_Code /METHOD=ENTER BMI_Code Anemia_History_Code Hypertension_Code Breastfeeding_Code Lifestyle_Code Supplement_Code Meat_Intake_Code /CRITERIA=PIN(0.05) POUT(0.10) ITERATE(20) CUT(0.5).
Resources	Processor Time	00:00:00.02
	Elapsed Time	00:00:00.02

Case Processing Summary

Unweighted Cases[a]		N	Percent
Selected Cases	Included in Analysis	100	100.0
	Missing Cases	0	.0
	Total	100	100.0
Unselected Cases		0	.0
Total		100	100.0

a. If weight is in effect, see classification table for the total number of cases.

Dependent Variable Encoding

Original Value	Internal Value
Negative	0
Positive	1

Block 0: Beginning Block

Classification Table[a,b]

Observed			Predicted: Iron Deficiency Status: Negative	Predicted: Iron Deficiency Status: Positive	Percentage Correct
Step 0	Iron Deficiency Status	Negative	59	0	100.0
		Positive	41	0	.0
	Overall Percentage				59.0

a. Constant is included in the model.

b. The cut value is .500

Variables in the Equation

		B	S.E.	Wald	df	Sig.	Exp(B)
Step 0	Constant	-.364	.203	3.204	1	.073	.695

Variables not in the Equation

			Score	df	Sig.
Step 0	Variables	BMI_Code	44.236	1	.000
		Anemia_History_Code	53.224	1	.000
		Hypertension_Code	38.253	1	.000
		Breastfeeding_Code	50.321	1	.000
		Lifestyle_Code	84.934	1	.000
		Supplement_Code	39.089	1	.000
		Meat_Intake_Code	44.429	1	.000
	Overall Statistics		87.139	7	.000

Block 1: Method = Enter

Omnibus Tests of Model Coefficients

		Chi-square	df	Sig.
Step 1	Step	116.302	7	.000
	Block	116.302	7	.000
	Model	116.302	7	.000

Model Summary

Step	-2 Log likelihood	Cox & Snell R Square	Nagelkerke R Square
1	19.069[a]	.687	.927

a. Estimation terminated at iteration number 20 because maximum iterations has been reached. Final solution cannot be found.

Classification Table[a]

Observed			Predicted: Iron Deficiency Status: Negative	Predicted: Iron Deficiency Status: Positive	Percentage Correct
Step 1	Iron Deficiency Status	Negative	55	4	93.2
		Positive	0	41	100.0
	Overall Percentage				96.0

a. The cut value is .500

Variables in the Equation

		B	S.E.	Wald	df	Sig.
Step 1[a]	BMI_Code	.000	13712.680	.000	1	1.000
	Anemia_History_Code	19.950	16408.711	.000	1	.999
	Hypertension_Code	.000	21026.429	.000	1	1.000
	Breastfeeding_Code	.000	25744.177	.000	1	1.000
	Lifestyle_Code	-22.456	11147.530	.000	1	.998
	Supplement_Code	.000	26884.905	.000	1	1.000
	Meat_Intake_Code	.000	32817.460	.000	1	1.000
	Constant	1.253	37615.380	.000	1	1.000

Variables in the Equation

		Exp(B)
Step 1[a]	BMI_Code	1.000
	Anemia_History_Code	461564247.1
	Hypertension_Code	1.000
	Breastfeeding_Code	1.000
	Lifestyle_Code	.000
	Supplement_Code	1.000
	Meat_Intake_Code	1.000
	Constant	3.500

a. Variable(s) entered on step 1: BMI_Code, Anemia_History_Code, Hypertension_Code, Breastfeeding_Code, Lifestyle_Code, Supplement_Code, Meat_Intake_Code.
