# Supplementary material for: Iron deficiency in non-pregnant women with normal hemoglobin: a cross-sectional analysis of risk factors and clinical implications
Source: Front Med (Lausanne). 2026 Jan 23;12:1700235. doi: 10.3389/fmed.2025.1700235 (PMC12908407; doi:10.3389/fmed.2025.1700235)
Supplement: Supplementary file 2 [file Table_1.DOCX]

**SUPPLEMENTARY MATERIALS**

**Table S1: Subgroup Analysis - Women with Breastfeeding History (n=58)**

**Table S2: Post-Hoc Power Analysis**

**Figure S1. CONSORT-style flow diagram of participant recruitment and enrollment.**

**Supplementary** **Table S1**

| Variable | Crude OR (95% CI) | p-value | Adjusted OR (95% CI) | p-value |
| --- | --- | --- | --- | --- |
| History of anemia (Yes) | 6.28 (1.89-20.84) | 0.003 | 5.19 (1.45-18.61) | 0.011 |
| Underweight BMI | 2.94 (0.54-15.97) | 0.211 | 3.12 (0.48-20.23) | 0.229 |
| Sedentary lifestyle | 1.52 (0.38-6.10) | 0.562 | 1.89 (0.32-11.23) | 0.485 |

**Supplementary Table S2**

Underweight BMI Subgroup (n=15):

Observed adjusted OR = 2.84

Effect size Cohen's h = 0.60

Power (α=0.05, two-tailed) = 0.62

The underweight subgroup was underpowered to detect the observed effect with standard statistical power; the findings should be interpreted as preliminary.


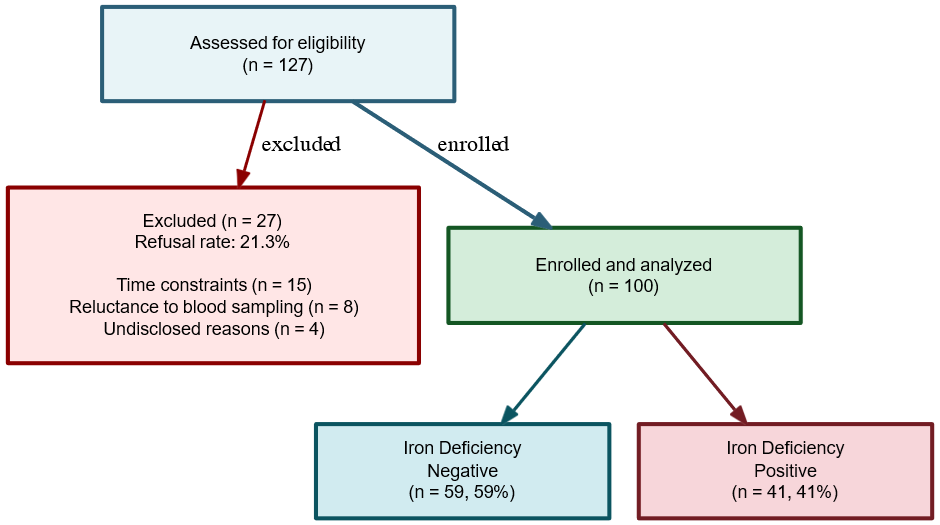


**Supplementary** **Figure S1.**

During the 6-month study period (March–August 2024), 127 non-pregnant women aged 16–45 years attending the outpatient department were assessed for eligibility. Twenty-seven women declined participation (refusal rate: 21.3%), with the majority citing time constraints (n=15, 55.6%), reluctance to provide blood samples (n=8, 29.6%), or undisclosed reasons (n=4, 14.8%).

One hundred women were enrolled and completed the study protocol with no dropouts or missing data. Final classification revealed 59 participants (59%) without iron deficiency and 41 participants (41%) with iron deficiency based on serum ferritin <15 µg/L or serum iron <10 µmol/L criteria.
